# Chromane Derivatives from Underground Parts of *Iris tenuifolia* and Their In Vitro Antimicrobial, Cytotoxicity and Antiproliferative Evaluation

**DOI:** 10.3390/molecules26216705

**Published:** 2021-11-05

**Authors:** Oldokh Otgon, Suvd Nadmid, Christian Paetz, Hans-Martin Dahse, Kerstin Voigt, Stefan Bartram, Wilhelm Boland, Enkhmaa Dagvadorj

**Affiliations:** 1 Department of Chemistry, School of Biomedicine, Mongolian National University of Medical Sciences, S. Zorig Street 3, Ulaanbaatar 14210, Mongolia; oldokh.o@mnums.edu.mn; 2 Department of Pharmaceutical Chemistry, School of Pharmacy, Mongolian National University of Medical Sciences, S. Zorig Street 3, Ulaanbaatar 14210, Mongolia; suvd.n@mnums.edu.mn; 3 Max Planck Institute for Chemical Ecology, Hans-Knoell-Strasse 8, D-07745 Jena, Germany; cpaetz@ice.mpg.de (C.P.); bartram@ice.mpg.de (S.B.); boland@ice.mpg.de (W.B.); 4 Leibniz Institute for Natural Product Research and Infection Biology, Hans Knöll Institute (HKI), Adolf-Reichwein-Strasse 23, D-07745 Jena, Germany; hans-martin.dahse@leibniz-hki.de (H.-M.D.); kerstin.voigt@hki-jena.de (K.V.)

**Keywords:** *Iris tenuifolia*, Iridaceae, chromane, macrolide, flavonoids, antimicrobial, antiproliferative effect, cytotoxicity

## Abstract

Phytochemical investigation of the ethanol extract of underground parts of *Iris tenuifolia* Pall. afforded five new compounds; an unusual macrolide termed moniristenulide (**1**), 5-methoxy-6,7-methylenedioxy-4-*O*-2′-cycloflavan (**2**), 5,7,2′,3′-tetrahydroxyflavanone (**3**), 5-hydroxy-6,7-dimethoxyisoflavone-2′-*O*-*β*-d-glucopyranoside (**9**), 5,2′,3′-dihydroxy-6,7-dimethoxyisoflavone (**10**), along with seven known compounds (**4**–**8**, **11**–**12**). The structures of all purified compounds were established by analysis of 1D and 2D NMR spectroscopy and HR-ESI-MS. The antimicrobial activity of the compounds **1**–**3**, **5**, **9**, and **10** was investigated using the agar diffusion method against fungi, Gram-positive and Gram-negative bacteria. In consequence, new compound **3** was found to possess the highest antibacterial activity against *Enterococcus faecalis* VRE and *Mycobacterium vaccae*. Cell proliferation and cytotoxicity tests were also applied on all isolated compounds and plant crude extract in vitro with the result of potent inhibitory effect against leukemia cells. In particular, the newly discovered isoflavone **10** was active against both of the leukemia cells K-562 and THP-1 while **4**–**6** of the flavanone type compounds were active against only THP-1.

## 1. Introduction

**Figure 1:**
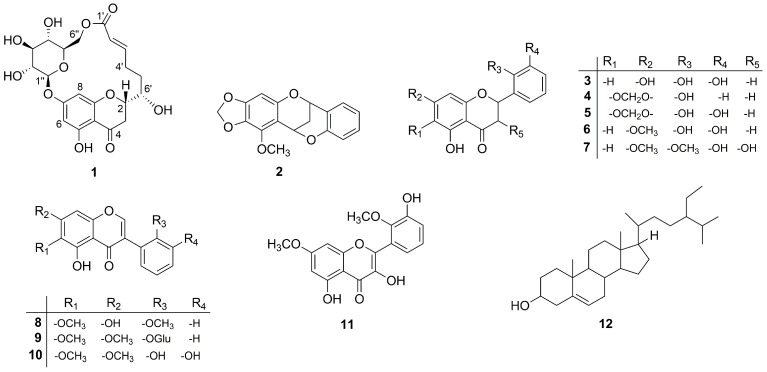
The structures of compounds **1–12**.

*Iris* is a genus of the family Iridaceae that consists of about 360 species of perennial herbs and sub shrubs. *Iris* species are widely distributed in the temperate northern hemisphere, including Euro-Asia, North Africa and North America [1]. Many members of the genus *Iris* have been used as traditional folk medicines for the treatment of various diseases, such as cancer, inflammation, bacterial and viral infections [2,3]. Previous phytochemical investigations on *Iris* plants have resulted in the isolation of a variety of secondary metabolites, which mainly include flavonoids, xanthones, terpenoids, steroids, quinones, stilbenes and simple phenolics. Moreover, the plant extracts and isolated compounds from these species were reported to have anti-inflammatory, antioxidant, antimicrobial, antitumor, antimutagenic, cytotoxic, immunomodulating, hepatoprotective, antiproliferative, estrogenic, cholinesterase inhibitory, cell proliferation stimulatory, antidiabetic and molluscicidal activities [1,4,5,6,7]. About 24 species are found in Mongolia. Specifically, *Iris tenuifolia* Pall., a characteristic plant of desert grasslands of Mongolia, is distributed in Mongol-Altai, Eastern Mongolia, Eastern Gobi and Gobi-Altai province [8,9]. The roots and rhizomes of *I. tenuifolia* are all commonly used as a traditional Mongolian medicine for treatment of kidney disorder. A root decoction of the plant has been used as a folk remedy for hypertension caused by adrenal gland diseases, ureter stones to relief renal colic and chronic nephritic [10]. This study deals with the isolation and structural elucidation of the new type of macrolide, named moniristenulide (**1**) and four previously unprecedented compounds: 5-methoxy-6,7-methylenedioxy-4-*O*-2′-cycloflavan (**2**), 5,7,2′,3′-tetrahydroxyflavanone (**3**), 5-hydroxy-6,7-dimethoxyisoflavone-2′-*O*-*β*-d-glucopyranoside (**9**), 5,2′,3′-trihydroxy-6,7-dimethoxyisoflavone (**10**), together with seven known compounds (Figure 1): four flavanone (**4**, **5**, **6**, **7**), one isoflavone (**8**), one flavonol (**11**), and one sterol (**12**). In addition, we aimed to evaluate the effectiveness and potency of these natural compounds using antimicrobial, cell proliferation and cytotoxicity assays.

## 2. Results and Discussion

### 2.1. Structure Elucidation

The ethanol extracts from the underground parts of *I. tenuifolia* were subjected to repeated column chromatography followed by crystallizations leading to the isolation of five unprecedented chromane derivatives.

**Figure 2:**
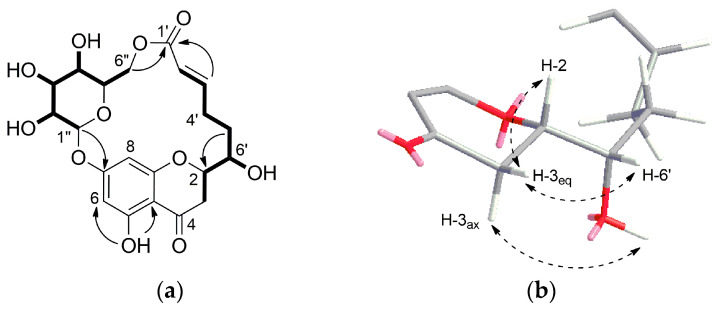
(**a**) ^1^H-^1^H COSY (**―**) and key HMBC (**→**) correlations of moniristenulide (**1**); (**b**) ROESY correlation (dashed arrow) establishing relative configuration of **1**.

Compound **1** was isolated as white crystal. HR-ESI-MS showed an ion peak at *m/z* 453.1409 [M + H]^+^ corresponding to a molecular formula of C_21_H_24_O_11_. Its ^1^H NMR spectrum acquired in DMSO-*d*_6_ (Table 1) showed resonances for meta-coupled aromatic protons at δ_H_ 6.14 (1H, *J* = 2.0 Hz) and δ_H_ 5.95 (1H, *J* = 2.0 Hz), two olefinic protons at δ_H_ 6.94 and δ_H_ 5.75, methylene signals between δ_H_ 3.21–1.81 and number of oxygenated protons between δ_H_ 5.50–3.08 corresponding to hydroxy groups as well as oxymethines. The presence of a 2,5,7-trisubstituted chromane-4-one was identified by analysis of HMBC correlations observed for the meta-coupled aromatic doublets H-6 and H-8 as well as methylene signals H-2 and H-3 (Figure 2a). Moreover, the ^1^H NMR spectrum exhibited a signal of one chelated hydroxyl group (δ_H_ 12.04), which is characteristic downfield shift of a hydroxyl group at C-5 and a carbonyl group at C-4. In addition, the presence of a hydroxyl group at C-5 was supported by HMBC correlations from 5-OH (δ_H_ 12.04) to C-5 (δ_C_ 163.1), C-6 (δ_C_ 97.3) and C-10 (δ_C_ 103.4). HSQC, HMBC, and COSY data clearly revealed the existence of a glucose residue. Further analysis of the spin-spin couplings (*J* = 7.7 Hz) allowed the identification of *β*-d-glucose. Connectivity of sugar moiety with chromane ring was deduced through HMBC correlation from the anomeric proton H-1″ (δ_H_ 5.09) to C-7 (δ_C_ 164.1) of aglycon. A remaining spin system observed on the COSY spectrum comprised a series of protons H-2′–H-6′. HMBC correlations from olefinic protons H-2′ and H-3′ to a carbonyl carbon at δ_C_ 164.8 (C-1′) allowed for establishing 6-hydroxy-2-hexenoic acid residue. *Trans* configuration of the double bond has been identified by a large coupling constant of 15.6 Hz. This substructure has been linked to the aglycon on position C-2 by HMBC correlations from the H-5′ and H-6′ to a carbon at δ_C_ 76.9 (C-2) and in turn H-3 to a carbon at δ_C_ 70.9 (C-6′). Interestingly, a macrolide structure was established via ester linkage between sugar residue and carbonyl carbon of hexenoic acid by strong heteronuclear long-range correlation from H-6″ to C-1′. ROESY correlations between diastereotopic H-3_eq_ (*β*) and H-2 and H-6′ indicated that these protons are on the same side of the chromane ring. In turn, H-3_ax_ showed ROESY correlation to 6′-OH. From the above findings, relative configuration at C-2 and C-6′ has been established as *β*-configured (Figure 2b). The absolute configuration at C-2 was found to be S as it showed positive and negative Cotton effects at 305 and 287 nm, respectively in the circular dichroism (CD) spectrum (Figure S9, Supplementary Materials) [11,12]. Thus, the structure of compound **1** was established as a new type of macrolide, named moniristenulide, as shown in Figure 1.

**Figure 3:**
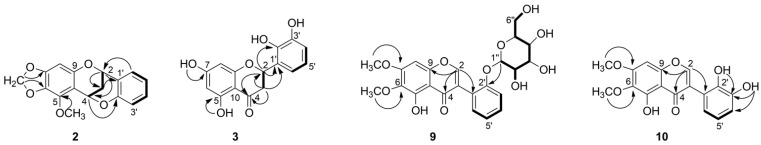
^1^H-^1^H COSY (**―**) and key HMBC (**→**) correlations of **2**, **3**, **9**, and **10**.

Compound **2** was isolated as colorless crystal. Its HR-ESI-MS at *m/z* 299.0919 [M + H]^+^ suggested a molecular formula of C_17_H_14_O_5_. Its ^1^H NMR spectrum showed resonances for five aromatic protons at δ_H_ 7.33 (1H, dd, *J* = 1.7, 7.5 Hz), 7.20 (1H, ddd, *J* = 1.7, 7.4, 7.4 Hz), 6.89 (1H, overlap.), 6.87 (1H, overlap.), 6.08 (1H, s), two signals of oxygenated methylene at δ_H_ 5.81 and δ_H_ 5.76 (each 1H, d, *J* = 1.5 Hz), two oxygenated methine protons at δ_H_ 5.64 (1H, dd, *J* = 2.8, 4.4 Hz) and 5.25 (1H, dd, *J* = 2.6, 4.4 Hz), two signals of diastereotopic methylene protons at δ_H_ 2.27 (1H, dt, *J* = 2.8, 13.8 Hz) and 2.15 (1H, dt, *J* = 2.8, 13.8 Hz) and one methoxy singlet at δ_H_ 4.09 (3H, s). Analysis of ^13^C NMR spectrum combined with HSQC allowed for identifying the existence of 17 carbon atoms, including 12 sp^2^ carbons, two methylene carbons at δ_C_ 26.5 and 100.8, two methine carbons at δ_C_ 67.5 and 62.4, and one methoxy group at δ_C_ 60.1 (Table 2). The COSY correlations of four aromatic protons at δ_H_ 7.33, 7.20, 6.89 and 6.87 (H-3′–H-6′) along with the respective HMBC correlations confirmed a presence of disubstituted aromatic B-ring, while the remaining sharp singlet at δ_H_ 6.08 (H-8) described a typical penta-substituted aromatic A-ring. Furthermore, the COSY experiment showed another spin system from H-2 to H-4 and indicated a CH-CH_2_-CH sequence. Additionally, the key HMBC correlation from H-6′ (δ_H_ 7.33) to C-2 (δ_C_ 67.5) revealed the flavan (2-phenylchromane) skeleton (Figure 3). The HMBC correlation from methine H-4 (δ_H_ 5.64) to C-2′ (δ_C_ 153.6) and their chemical shift indicated their linkage through an oxygen bridge. All these data deduced that compound **2** has a 4-*O*-2′-cycloflavan as a partial structure. The remaining two doublet signals at δ_H_ 5.81 and 5.76 had one bond correlation to a carbon atom at δ_C_ 100.8, which were assigned as a methylenedioxy group. These methylenedioxy doublet was connected to a flavan core by key HMBC correlations to C-6 and C-7. The location of methoxy group (δ_H_ 4.09) at C-5 was suggested by HMBC correlations. On the basis of the above evidence, the planar structure of compound **2** was elucidated as 5-methoxy-6,7-methylenedioxy-4-*O*-2′-cycloflavan and considered as an unprecedented natural product. This spectroscopic data of compound **2** is somewhat similar to the literature values for the known 4-*O*-2′-cycloflavan core structures, possessing a different substitution pattern in aromatic A- and B-rings [7,13,14].

Compound **3** was isolated as yellow solid. It showed a molecular ion peak at *m/z* 289.0714 [M + H]^+^ ascribable to a molecular formula of C_15_H_12_O_6_. The ^1^H NMR spectrum showed resonances for five aromatic proton signals at δ_H_ 5.88 (1H, d, *J* = 2.0 Hz), 5.90 (1H, d, *J* = 2.0 Hz), 6.69 (1H, t, *J* = 7.8 Hz), 6.79 (1H, dd, *J* = 1.4, 7.9 Hz), 6.88 (1H, dd, *J* = 1.4, 7.8 Hz), three aliphatic protons at δ_H_ 5.70 (1H, dd, *J* = 2.9, 12.9 Hz), 3.17 (1H, dd, *J* = 12.9, 17.0 Hz), and 2.69 (1H, dd, *J* = 3.0, 17.0 Hz), and four oxygenated protons at δ_H_ 12.12, 10.79, 9.51, and 8.71, one of which was appeared as a chelated hydroxyl group. The ^13^C NMR spectrum contained signals from 15 carbon atoms, which were with the full agreement of HR-MS (Table 2). Partial structure 2,5,7-trisubstituted chromane-4-one was deduced from the analysis of protons H-2 and H-3, which were existing in an AMX spin system (Figure 3). In addition, hydroxyl groups at C-5 and C-7 were supported by HMBC correlations from 5-OH (δ_H_ 12.12) to C-5 (δ_C_ 163.5), C-6 (δ_C_ 95.8) and C-10 (δ_C_ 101.7) and from 7-OH to C-6 (δ_C_ 95.8) and C-7 (δ_C_ 166.6). In addition, a COSY correlation of three aromatic protons at δ_H_ 6.69, 6.79, 6.88 along with HMBC correlations from proton H-4′ (δ_H_ 6.79) to C-2′ (δ_C_ 142.6), C-3′ (δ_C_ 145.2) and from proton H-6′ (δ_H_ 6.88) to C-2 (δ_C_ 74.0), C-2′ (δ_C_ 142.6), and C-4′ (δ_C_ 115.2) revealed a presence of a 2,3-dihydroxyphenyl moiety (ring-B) and altogether confirmed the flavanone structure. This was further supported by key long-range heteronuclear correlations from the methine proton H-2 (δ_H_ 5.70) to C-2′ (δ_C_ 142.6). The position of the remaining two hydroxyl protons at δ_H_ 9.51 and 8.71 were assigned to C-3′ and C-2′ respectively, due to observed HMBC correlations. Spectral data of **3** possess close similarity to those for the known compounds **5** and **6** [15]. The only difference was observed for the substitution on C-7, where methoxy group in **6** and methylenedioxy group in **5,** while it was replaced by hydroxy group in **3**. This was supported by HMBC correlations from 7-OH to C-6, C-7, and C-8 in **3** (Figure 3).

The absolute configuration at C-2 of compound **3** was determined as 2S based on its CD spectrum (Figure S25, Supplementary Materials), which displayed a positive Cotton effect at 325 nm and a negative one at 283 nm [11,12]. Consequently, compound **3** was elucidated as (2S)-5,7,2′,3′-tetrahydroxyflavanone, an undescribed member of a flavanone group of natural products.

Compound **9** was isolated as yellow crystal. The molecular formula C_23_H_24_O_11_ was established on the basis of the positive ion at *m/z* 477.1399 [M + H]^+^ by HR-ESI-MS. The ^1^H NMR spectrum revealed the presence of five aromatic protons (Table 2). The COSY spectrum showed a spin system comprising four aromatic protons at δ_H_ 7.37 (1H, overlap.), 7.27 (1H, d, *J* = 8.2 Hz), 7.35 (1H, overlap.), and 7.09 (1H, t, *J* = 7.5 Hz), characteristic for an *ortho*-substituted B-ring of aglycone (Figure 3). The remaining aromatic signal appeared as a sharp singlet at 6.64 (1H, s) together with chelated hydroxyl group at δ_H_ 12.68 suggested a typical penta-substituted aromatic A-ring. The singlet was assigned on C-8 according to HMBC correlation from H-8 to C-9 (δ_C_ 149.2) and C-7 (δ_C_ 158.3). The chelated hydroxyl group was positioned at C-5 by means of HMBC. In addition, a characteristic isoflavonoid signal for H-2 was appeared at δ_H_ 8.44. The isoflavone nature was supported by long-range correlations from H-2 (δ_H_ 8.44) to C-4 (δ_C_ 180.5), C-9 (δ_C_ 149.2), and C-1′ (δ_C_ 120.0) in the HMBC spectrum. Furthermore, two methoxyl singlet signals were apparent at δ_H_ 3.79 and 3.94, and they were located at C-6 and C-7 due to HMBC correlations between 6-OCH_3_ and C-6 (δ_C_ 128.3), as well as 7-OCH_3_ and C-7 (δ_C_ 158.3). Moreover, a series of COSY cross signals comprising six protons in the range of δ_H_ 3.0–4.0, four hydroxy protons δ_H_ 4.57–5.05, as well as a doublet at δ_H_ 4.89, revealed the presence of a glucose moiety (H-1″ to H-6″) [16]. The HMBC correlation from H-1″ (δ_H_ 4.89) to C-2′ (δ_C_ 155.1) revealed the sugar moiety was located at C-2′ of aglycone. The coupling constant of anomeric proton *J* = 7.80 Hz indicated that the sugar was *β*-oriented. The ^1^H and ^13^C NMR spectroscopic data of aglycone of **9** were comparable with the literature values for the irilin A [17,18]. The only difference was occurred on C-2′, where OH group of irilin A was replaced by glucopyranosyl in **9**. Finally, the structure of compound **9** was elucidated as 5-hydroxy-6,7-dimethoxyisoflavone-2′-*O*-*β*-d-glucopyranoside, an unprecedented natural product.

Compound **10** was isolated as yellow crystal. HR-ESI-MS showed the [M + H]^+^ peak at *m/z* 331.0825, corresponding to the molecular formula C_17_H_14_O_7_. ^1^H NMR, ^13^C NMR, and HSQC data of compound **10** closely resembled to that of **9**, differing only on the absence of signals corresponding to a sugar moiety and different pattern on aromatic signals of B-ring (Table 2). The COSY correlation of a triplet at δ_H_ 6.92 (1H, t, *J* = 7.9 Hz) with two doublets of doublets at δ_H_ 7.03 (1H, dd, *J* = 1.3, 7.9 Hz) and 6.71 (1H, dd, *J* = 1.5, 7.8 Hz), and their HMBC correlations clearly indicated *ortho*-dihydroxyl substitution on B-ring. The position of the hydroxyl groups, which were appeared as two singlets in the ^1^H NMR spectrum at δ_H_ 8.48 and 6.09, were assigned at C-2′ and C-3′, due to the HMBC correlations (Figure 3). Therefore, the structure of **10** was solved as 5,2′,3′-trihydroxy-6,7-dimethoxyisoflavone, which is an undescribed natural product so far.

Furthermore, the known compounds were readily identified by means of HR-ESI-MS, 1D, and 2D NMR data as well as in comparison with those previously reported in the literature: compound **4** as 5,2′-dihydroxy-6,7-methylenedioxyflavanone [15], **5** as 5,2′,3′-trihydroxy-6,7-methylenedioxyflavanone [15], **6** as 5,2′,3′-trihydroxy -7-methoxyflavanone [15], **7** as 3,5,3′-trihydroxy-7,2′-dimethoxyflavanone [15], **8** as 5,7-dihydroxy-6,2′-dimethoxyisoflavone [19], **11** as 3,5,3′-trihydroxy-7,2′-dimethoxy -flavonol [20], and **12** as *β*-sitosterol [2,21] (Figure 1). To the best of our knowledge, the known compound **11** was isolated for the first time from this plant.

### 2.2. Antimicrobial Activity

The newly discovered compounds **1**, **2**, **3**, **9**, and **10** were tested together with the known compound **5**, which is the major component of the roots of this plant [15] for their antifungal and antibacterial activities against three fungi and eight bacterial strains (Table 3) using the agar diffusion method. All tested compounds possessed weak to moderate activity against vancomycin resistant (VRE) *Enterococcus faecalis*. Compounds except **1** and **9** exhibited weak and moderate activities against *Bacillus subtilis* and *Mycobacterium vaccae*, respectively, compared to ciprofloxacin. Compounds **3**, **5**, and **10**, which contain *ortho*-dihydroxyl groups in B-ring at positions 2′ and 3′, were most active against bacterial strains. This supports the evidence that the *ortho* -dihydroxyl structural fragment in B-ring is important for antimicrobial activity [22]. Compound **2**, a new cycloflavan, demonstrated activity against *B. subtilis*, *E. faecalis*, and *M. vaccae*. Interestingly, the new compounds **1** and **9**, which have a glucose unit in their structure, showed selective activity only against *E. faecalis* VRE.

As for the fungi, the compound **5** showed moderate activity against *Penicillium notatum*, and compounds **5** and **10** showed weak activity against *Candida albicans*. It is noteworthy that compound **5**, which contains a methylenedioxy group, in addition to the *ortho*-dihydroxyl groups in B-ring, showed broad activity against eight microorganisms out of eleven. However, no inhibition was observed with the tested compounds against bacterial strains of *Escherichia coli* and *Pseudomonas aeruginosa* (SG137 B7) or against the fungus *Sporobolomyces Salmonicolor*. This is the first report on the antimicrobial activity of the tested compounds.

### 2.3. Antiproliferative and Cytotoxic Activities

Using HUVEC, K-562, THP-1, A549, and HeLa cell lines, the antiproliferative activities and the cytotoxicity of the isolated compounds (**1**–**12**) along with plant raw extract were evaluated in vitro (Table 4). The plant extract possessed antiproliferative effects against leukemia cell lines and a cytotoxic effect on Hela cells. With the exception of **12**, the compounds tested in this study were chromane derivatives. Compounds **4**–**6** and **10** showed inhibition effects against all of the applied cancer cell lines and exhibited potential antiproliferative activities against the THP-1 cell line with GI_50_ values at 16.0, 16.5, 16.9, and 9.1 µM, respectively. Moreover, compound **10** demonstrated the most potent activity (GI_50_ = 7.6 µM) against the K-562, followed by compounds **2** and **3** with GI_50_ value at 31.5 and 32.3 µM. Compound **3**, the tetrahydroxy flavanone, showed selective antiproliferative inhibition effect against the K-562 cells only. These results are in a good agreement with previous studies and supports the evidence that the *ortho*-dihydroxyl structural fragment in B-ring is very important for anticancer activity [7,23,24,25]. Furthermore, compound **2**, the cycloflavan, exhibited significant inhibitory activities against cell lines HUVEC and THP-1 with a GI_50_ values at 35.2 and 29.2 µM and showed the cytotoxic effect on HeLa cells with CC_50_ value at 42.6 µM among the tested compounds. Besides that, compound **10** showed a similar moderate inhibition effect (GI_50_ = 35.8 µM) on HUVEC cells. All the flavonoids examined showed their low toxicity with CC_50_ values of more than 100 μM on HeLa cells. In cases of compounds **7** and **8** which do not have *ortho*-dihydroxy substitutions on the A and B rings, reduced or no inhibitory effects were found compared to other compounds mentioned above. Compound **11** showed slightly increased activity against K-562 and moreover against THP-1 and A549 cell lines compared to compound **7**. Thus, it supports the relevance of the C-2 and C-3 double bond in a flavonoid structure [23]. In contrast to **10**, compound **9**, which differs in its structure in the presence of a sugar moiety and a dehydroxylation on the B-ring, became completely not effective. Interestingly, compound **1**, which also has a sugar residue in its structure, did not show any activity up to 100 µM against all human cancer cell lines either. Hence, the results are consistent with previous research that flavonoids glycosides were generally not effective against multiple cancer cell lines [23]. Compound **12** showed neither an antiproliferative effect nor a cytotoxicity within the tested range against all applied cell lines.

## 3. Materials and Methods

### 3.1. General Experimental Procedures

Solvents and reagents were purchased from Sigma-Aldrich, Deisenhofen, Germany and Qingdao Marine Chemical, China. The optical data were measured using a digital JASCO P-2000 polarimeter (Jasco, Pfungstadt, Germany). The CD spectrum was recorded on a JASCO J810 spectropolarimeter (Jasco, Pfungstadt, Germany). Ultraviolet-visible (UV-Vis) data were extracted from diode array detector (DAD) data obtained during high performance liquid chromatography-electrospray ionization-high resolution mass spectrometry (HPLC-ESI-HRMS) experiments.

NMR spectra were recorded at 298 K on 500 MHz Bruker Avance III HD spectrometer (Bruker Biospin, Rheinstetten, Germany), equipped with cryoplatforms and TCI cryoprobes (5 mm). Spectrometer control and data processing were accomplished using Bruker Topspin ver.3.2 (Bruker Biospin, Rheinstetten, Germany), and standard pulse programs were used. NMR signals were referenced to the respective solvent signals at δ_H_ 2.50 and δ_C_ 39.53 for hexadeuterodimethyl sulfoxide DMSO-d_6_ and δ_H_ 7.26 and δ_C_ 77.06 for deuterochloroform CDCl_3_. HPLC-ESI-HRMS spectra were recorded on an Agilent Infinity 1260, consisting of a combined degasser and quaternary pump, column oven, autosampler, and DAD. The DAD was coupled to a Bruker Compact quadrupole time-of-flight (QTOF) mass spectrometer (Bruker Daltonics, Bremen, Germany). Both devices were controlled by Bruker Compass ver.1.9 (Bruker Daltonics, Bremen, Germany). For HPLC separation, an Agilent Zorbax C-18 SB column (3.5 µm, 4.6 × 150 mm i.d.) was used. The mass spectrometer was operated, depending on the analyte, either in positive or negative ionization mode, employing an electrospray ionization (ESI) source. The standard settings for small molecule analysis, as provided with Bruker Compass, were used. Column chromatographic separations were performed on silica gel (200–300 mesh, Merck, Darmstadt, Germany and Qingdao Marine Chemical, China) and Sephadex LH-20 (Pharmacia Fine Chemical, Uppsala, Sweden). Thin-layer chromatography (TLC) was performed on pre-coated TLC plates with silica gel 60 F_254_ (Merck, Darmstadt, Germany). Spots were detected under UV absorption (λ_max_ 254 and 364 nm) by spraying with 1% methanolic diphenylboric acid-*β*-ethylaminoester, 5% ethanolic polyethylene glycol or under visible light by spraying with 5% ethanolic sulfuric acid and 1% acidified methanolic vanillin.

### 3.2. Plant Material

Rhizomes and roots of *I. tenuifolia* were collected in September 2016 from Khurmen Sum of South Gobi province of Mongolia. It was identified by Urgamal Magsar, a botanist of the Institute of General and Experimental Biology, Mongolian Academy of Sciences, where voucher specimens (It 0916) of the plant have been deposited.

### 3.3. Extraction and Isolation

The air dried and powdered plant material (5.0 kg) was extracted three times with 95% ethanol (crude extract I) and three more times with 50% ethanol (crude extract II). The extracts were evaporated under vacuum to yield a brown residue. Crude extract I (700 g) was fractionated by column chromatography on a silica gel column, eluted with dichloromethane (CH_2_Cl_2_) and mixtures of CH_2_Cl_2_ and methanol (CH_2_Cl_2_-MeOH) (50:1, 30:1, 10:1, 5:1, 1:1, *v*/*v*) with increasing polarity. Eluates were pooled into seven fractions (A–G) on the basis of TLC analysis. Fractions A and B were subjected to column chromatography on silica gel eluted with a chloroform (CHCl_3_) and the mixture of CHCl_3_-MeOH (70:1, 50:1, 30:1, 10:1, 5:1, 1:1, *v/v*) with increasing polarity to give subfractions A.1–A.7 and B.1–B.6, respectively. Subfraction A.2 was further purified on silica gel column eluting with petroleum ether and ethyl acetate (pet.ether-EtOAc) (50:1, 30:1, 10:1, 5:1, 1:1, *v*/*v*) followed by Sephadex LH-20 eluting with CHCl_3_-MeOH (1:1, *v*/*v*) to yield compounds **4** (20.4 mg), **7** (39.9 mg), **8** (328.8 mg), **10** (85.9 mg), **11** (29.3 mg), and **12** (151.8 mg). Further subfraction B.5 was subjected to silica gel column chromatography eluting with CHCl_3_-MeOH (70:1, 50:1, 30:1, 10:1, 5:1, 3:1, 1:1, *v/v*) followed by Sephadex LH-20 with CHCl_3_-MeOH (1:1, *v/v*) to yield compounds **3** (105.6 mg), **5** (88.2 mg), and **6** (19.4 mg). These compounds were recrystallized from CHCl_3_-MeOH (1:1, *v*/*v*). Fraction D was separated on silica gel column chromatography using CHCl_3_-MeOH (50:1, 30:1, 10:1, 5:1, 1:1, *v/v*) as an eluent to give eight subfractions (D.1–D.8). Compound **9** (11.5 mg) was obtained in pure form by recrystallisation from CHCl_3_-MeOH (1:1 *v/v*) from subfraction D.4. Subfraction D.5 was further subjected on silica gel eluting with CHCl_3_-MeOH (10:1, *v/v*) and then purified on silica gel with pet.ether-EtOAc (5:1, 3:1, 1:1, 1:3, 1:5, *v/v*) to afford compound **1** (6.9 mg). Crude extract II (52 g) was fractionated by column chromatography on silica gel eluted with CHCl_3_ and CHCl_3_-MeOH (CHCl_3_, 50:1, 30:1, 10:1, 5:1, 3:1, 1:1, MeOH, *v/v*) and pooled into eight fractions (A–H). Fraction A was subjected to silica gel column eluted with a gradient of pet.ether-EtOAc (50:1, 30:1, 10:1, 5:1, 1:1, *v/v*) to yield compound **2** (11.0 mg) and which was further recrystallized from CHCl_3_-MeOH (1:1, *v/v*).

Moniristenulide (**1**): white powder, [α]_D_^25^ + 44 (*c* = 0.18, DMSO), CD (*c* = 0.22, MeOH) 305 nm (Δε + 1.25) and 287 nm (Δε − 3.96), UV (CH_3_CN/H_2_O): λ_max_ 206, 228, 282, 326 nm; ^1^H NMR (500 MHz, DMSO-*d*_6_) and ^13^C NMR (125 MHz, DMSO-*d*_6_) data, see Table 1; HR-ESI-MS: *m/z* 453.1409 [M + H]^+^ (calcd. for C_21_H_25_O_11_, 453.1397).

5-Methoxy-6,7-methylenedioxy-4-*O*-2′-cycloflavan (**2**): colorless crystal, [α]_D_^25^ + 317 (*c* = 0.67, DMSO), UV (CH_3_CN/H_2_O): λ_max_ 196, 206, 286 nm; ^1^H NMR (500 MHz, CDCl_3_) and ^13^C NMR (125 MHz, CDCl_3_) data, see Table 2; HR-ESI-MS: *m/z* 299.0919 [M + H]^+^ (calcd. for C_17_H_15_O_5_, 299.0920).

(2S)-5,7,2′,3′-Tetrahydroxyflavanone (**3**): yellow powder; [α]_D_^25^ + 10 (*c* = 0.67, DMSO), CD (*c* = 0.69, MeOH) 325 nm (Δε + 2.40) and 283 nm (Δε − 11.70), UV (CH_3_CN/H_2_O): λ_max_ 202, 288 nm; ^1^H NMR (500 MHz, DMSO-*d*_6_) and ^13^C NMR (125 MHz, DMSO-d_6_) data, see Table 2; HR-ESI-MS: *m/z* 289.0714 [M + H]^+^ (calcd. for C_15_H_13_O_6_, 289.0712).

5,2′-Dihydroxy-6,7-methylenedioxyflavanone (**4**): yellow powder, UV (CH_3_CN/H_2_O): λ_max_ 206, 228, 280 nm; ^1^H NMR (500 MHz, DMSO-*d*_6_) δ_H_ 11.86 (1H, s, 5-OH), 9.89 (1H, s, 2′-OH), 7.43 (1H, d, *J* = 7.5 Hz, H-6′), 7.19 (1H, t, *J* = 7.5 Hz, H-4′), 6.88 (1H, overlap, H-5′), 6.86 (1H, overlap, H-3′), 6.31 (1H, s, H-8), 6.06 (2H, d, *J* = 6.9 Hz, -CH_2_-), 5.72 (1H, dd, *J* = 2.8, 13.1 Hz, H-2), 3.26 (1H, dd, *J* = 13.4, 17.1 Hz, H-3a), 2.74 (1H, dd, *J* = 2.9, 17.1 Hz, H-3b). ^13^C NMR (125 MHz, DMSO-*d*_6_) δ_C_ 74.6 (C-2), 41.2 (C-3), 197.9 (C-4), 143.1 (C-5), 127.4 (C-6), 155.8 (C-7), 90.4 (C-8), 159.4 (C-9), 103.6 (C-10), 124.5 (C-1′), 154.4 (C-2′), 115.6 (C-3′), 129.6 (C-4′), 119.2 (C-5′), 127.1 (C-6′), 102.5 (-CH2-). HR-ESI-MS: *m/z* 301.0722 [M + H]^+^ (calcd. for C_16_H_13_O_6_, 301.0712).

5,2′,3′-Trihydroxy-6,7-methylenedioxyflavanone (**5**): yellow crystal, [α]_D_^25^ + 9 (*c* = 0.8, DMSO), UV (CH_3_CN/H_2_O): λ_max_ 206, 244, 284 nm; ^1^H NMR (500 MHz, DMSO-*d*_6_) δ_H_ 11.88 (1H, s, 5-OH), 9.52 (1H, s, 3′-OH), 8.74 (1H, s, 2′-OH), 6.90 (1H, d, *J* = 7.8 Hz, H-6′), 6.80 (1H, dd, *J* = 1.2, 7.8 Hz, H-4′), 6.69 (1H, t, *J* = 7.8 Hz, H-5′), 6.30 (1H, s, H-8), 6.07 (2H, d, *J* = 6.6 Hz, -CH2-), 5.73 (1H, dd, *J* = 2.8, 13.2 Hz, H-2), 3.24 (1H, dd, *J* = 13.3, 17.2 Hz, H-3a), 2.73 (1H, dd, *J* = 2.9, 17.2 Hz, H-3b). ^13^C NMR (125 MHz, DMSO-*d*_6_) δ_C_ 74.6 (C-2), 41.2 (C-3), 197.9 (C-4), 143.1 (C-5), 127.3 (C-6), 155.7 (C-7), 90.4 (C-8), 159.4 (C-9), 103.6 (C-10), 125.2 (C-1′), 142.6 (C-2′), 145.2 (C-3′), 115.3 (C-4′), 119.0 (C-5′), 117.1 (C-6′), 102.4 (-CH_2_-). HR-ESI-MS: *m/z* 317.0666 [M + H]^+^ (calcd. for C_16_H_13_O_7_, 317.0661).

5,2′,3′-Trihydroxy-7-methoxyflavanone (**6**): yellow crystal; UV (CH_3_CN/H_2_O): λ_max_ 206, 226, 286 nm; ^1^H NMR (500 MHz, DMSO-*d*_6_) δ_H_ 12.09 (1H, s, 5-OH), 9.56 (1H, s, 3′-OH), 8.74 (1H, s, 2′-OH), 6.89 (1H, d, *J* = 7.7 Hz, H-6′), 6.79 (1H, d, *J* = 7.7 Hz, H-4′), 6.69 (1H, t, *J* = 7.8 Hz, H-5′), 6.11 (1H, d, *J* = 2.0 Hz, H-8), 6.08 (1H, d, *J* = 2.0 Hz, H-6), 5.73 (1H, dd, *J* = 2.8, 12.9 Hz, H-2), 3.79 (3H, s, 7-OCH_3_), 3.23 (1H, dd, *J* = 12.9, 17.2 Hz, H-3a), 2.73 (1H, dd, *J* = 3.0, 17.2 Hz, H-3b). ^13^C NMR (125 MHz, DMSO- *d*_6_) δ_C_ 74.2 (C-2), 41.2 (C-3), 196.9 (C-4), 163.2 (C-5), 94.7 (C-6), 167.4 (C-7), 93.8 (C-8), 159.4 (C-9), 102.6 (C-10), 125.4 (C-1′), 143.1 (C-2′), 145.2 (C-3′), 115.2 (C-4′), 119.0 (C-5′), 117.1 (C-6′), 55.9 (7-OCH_3_). HR-ESI-MS: *m/z* 303.0877 [M + H]^+^ (calcd. for C_16_H_15_O_6_, 303.0868).

3,5,3′-Trihydroxy-7,2′-dimethoxyflavanone (**7**): white powder; UV (CH_3_CN/H_2_O): λ_max_ 206, 226, 282 nm; ^1^H NMR (500 MHz, DMSO- *d*_6_) δ_H_ 11.88 (1H, s, 5-OH), 9.49 (1H, s, 3′-OH), 6.99 (1H, overlap, H-6′), 6.97 (1H, overlap, H-5′), 6.90 (1H, dd, *J* = 3.1, 6.3 Hz, H-4′), 6.13 (1H, d, *J* = 2.2 Hz, H-6), 6.08 (1H, d, *J* = 2.2 Hz, H-8), 5.94 (1H, d, *J* = 6.1 Hz, 3-OH), 5.46 (1H, d, *J* = 11.6 Hz, H-2), 4.77 (1H, dd, *J* = 6.1, 11.5 Hz, H-3), 3.78 (3H, s, 7-OCH_3_), 3.74 (3H, s, 2′-OCH_3_). ^13^C NMR (125 MHz, DMSO- *d*_6_) δ_C_ 77.7 (C-2), 70.7 (C-3), 198.4 (C-4), 163.1 (C-5), 94.9 (C-6), 167.6 (C-7), 93.8 (C-8), 162.5 (C-9), 101.4 (C-10), 130.4 (C-1′), 146.7 (C-2′), 150.2 (C-3′), 117.2 (C-4′), 123.9 (C-5′), 118.6 (C-6′), 60.5 (7-OCH_3_), 55.9 (2′-OCH_3_). HR-ESI-MS: *m/z* 333.0982 [M + H]^+^ (calcd. for C_17_H_17_O_7_, 333.0974).

5,7-Dihydroxy-6,2′-dimethoxyisoflavone (**8**): yellow crystal, UV (CH_3_CN/H_2_O): λ_max_ 196, 226, 262 nm; ^1^H NMR (500 MHz, DMSO-*d*_6_) δ_H_ 12.94 (1H, s, 5-OH), 10.78 (1H, s, 7-OH), 8.24 (1H, s, H-2), 7.40 (1H, dd, *J* = 1.4, 7.5 Hz, H-4′), 7.24 (1H, dd, *J* = 1.4, 7.5 Hz, H-6′), 7.09 (1H, d, *J* = 8.3 Hz, H-3′), 7.00 (1H, t, *J* = 7.5 Hz, H-5′), 6.52 (1H, s, H-8), 3.75 (3H, s, 6-OCH_3_), 3.73 (3H, s, 2′-OCH_3_). ^13^C NMR (125 MHz, DMSO-*d*_6_) δ_C_ 155.3 (C-2), 119.8 (C-3), 180.1 (C-4), 152.8 (C-5), 131.5 (C-6), 157.5 (C-7), 94.0 (C-8), 153.1 (C-9), 104.7 (C-10), 120.2 (C-1′), 157.5 (C-2′), 111.3 (C-3′), 129.9 (C-4′), 120.0 (C-5′), 131.6 (C-6′), 59.9 (6-OCH_3_), 55.6 (7-OCH_3_). HR-ESI-MS: *m/z* 315.0888 [M + H]^+^ (calcd. for C_17_H_15_O_6_, 315.0868).

5-Hydroxy-6,7-dimethoxyisoflavone-2′-*O*-*β*-d-glucopyranoside (**9**): yellow crystal, UV (CH_3_CN/H_2_O): λ_max_ 206, 216, 262, 336 nm; ^1^H NMR (500 MHz, DMSO-*d*_6_) and ^13^C NMR (125 MHz, DMSO-*d*_6_) data, see Table 2; HR-ESI-MS: *m/z* 477.1399 [M + H]^+^ (calcd. for C_23_H_25_O_11_, 477.1397)

5,2′,3′-Trihydroxy-6,7-dimethoxyisoflavone (**10**): yellow powder, UV (CH_3_CN/H_2_O): λ_max_ 206, 222, 258, 338 nm; ^1^H NMR (500 MHz, CDCl_3_) and ^13^C NMR (125 MHz, CDCl_3_) data, see Table 2; HR-ESI-MS: *m/z* 331.0825 [M + H]^+^ (calcd. for C_17_H_15_O_7_, 331.0818).

3,5,3′-Trihydroxy-7,2′-dimethoxyflavonol (Irisflavone D) (**11**): yellow amorphous powder, UV (CH_3_CN/H_2_O): λ_max_ 198, 256, 302, 344 nm; ^1^H NMR (500 MHz, DMSO-*d*_6_) δ_H_ 12.48 (1H, s, 5-OH), 9.70 (1H, s, 3′-OH), 9.14 (1H, s, 3-OH), 7.03 (1H, overlap, H-5′), 7.02 (1H, overlap, H-4′), 6.93 (1H, t, *J* = 4.8 Hz, H-6′), 6.61 (1H, d, *J* = 2.1 Hz, H-8), 6.38 (1H, d, *J* = 2.1 Hz, H-6), 3.84 (3H, s, 7-OCH_3_), 3.77 (3H, s, 2′-OCH_3_). ^13^C NMR (125 MHz, DMSO- *d*_6_) δ_C_: 148.3 (C-2), 137.3 (C-3), 176.5 (C-4), 160.7 (C-5), 97.5 (C-6), 165.0 (C-7), 92.0 (C-8), 156.7 (C-9), 104.6 (C-10), 124.9 (C-1′), 145.9 (C-2′), 150.5 (C-3′), 123.7 (C-4′), 118.8 (C-5′), 120.9 (C-6′), 60.3 (2′-OCH_3_), 56.1 (7-OCH_3_). HR-ESI-MS: *m/z* 331.0835 [M + H]^+^ (calcd. for C_17_H_15_O_7_, 331.0817).

*β*-Sitosterol (**12**): white powder, ^1^H NMR (500 MHz, CDCl_3_) δ_H_ 5.35 (1H, d, *J* = 5.3 Hz, H-5), 3.52 (1H, m, H-3), 1.01 (3H, s, H-29), 0.92 (3H, d, *J* = 6.62 Hz, H-19), 0.84 (3H, t, *J* = 7.5 Hz, H-24), 0.82 (3H, d, *J* = 1.8 Hz, H-26), 0.80 (3H, d, *J* = 7.0 Hz, H-27), 0.68 (3H, s, H-28). ^13^C NMR (CDCl_3_, 125 MHz) δ_C_ 37.4 (C-1), 31.8 (C-2), 71.9 (C-3), 42.5 (C-4), 140.9 (C-5), 121.9 (C-8), 29.8 (C-7), 32.0 (C-8), 50.3 (C-9), 36.6 (C-10), 21.2 (C-11), 39.9 (C-12), 42.4 (C-13), 56.9 (C-14), 25.5 (C-15), 28.4 (C-16), 56.2 (C-17), 36.3 (C-18), 19.2 (C-19), 34.1 (C-20), 26.2 (C-21), 45.9 (C-22), 23.2 (C-23), 12.1 (C-24), 29.3 (C-25), 19.9 (C-26), 19.5 (C-27), 18.9 (C-28), 12.0 (C-29).

### 3.4. Antimicrobial Activity Assay

Compounds **1**, **2**, **3**, **5**, **9**, and **10** were tested for their antimicrobial activities against *S. aureus* (JMRC:STI 10760), *B. subtilis* (JMRC:STI 10880), *S. aureus* MRSA (JMRC: ST 33793) *E. faecalis* VRE (JMRC: ST 33700), *E. coli* (JMRC:ST 33699), *P. aeruginosa* (JMRC:ST 33771), *P. aeruginosa* (JMRC:ST 33772), *M. vaccae* (JMRC:STI 10670), *S. salmonicolor* (JMRC:ST 35974), *C. albicans* (JMRC:STI 25000), and *P. notatum* (JMRC:STI 50164)) using agar diffusion assay as previously published [26]. Strains were obtained from the Jena Microbial Resource Collection (JMRC). The bacteria were cultivated on standard I nutrient agar in Petri dishes at 37 °C. Antifungal bioassays were conducted at 30 °C using the basidiomycetous yeast *S. salmonicolor* and the filamentous ascomycete *P. notatum*, which were cultivated on malt agar, and the ascomycetous yeast *C. albicans*, which was cultivated on yeast morphology agar. After inoculation of the test organisms, a disc (9 mm in diameter) was removed from the center of the Petri dish and 50 μL of the test solution (1 mg/mL in DMSO) was added to the cavity. After 18 h of incubation, the inhibiting areola were measured and documented as diameters in mm. Ciprofloxacin (5 µg/mL in deionized water) and amphotericin B (10 µg/mL in DMSO/MeOH 1:1) were used as reference substances against bacterial and fungal strains, respectively.

### 3.5. Antiproliferation and Cytotoxicity Assays

Compounds (**1**–**12**) were assayed against human umbilical vein endothelial cells (HUVEC), human chronic myeloid leukemia cells (K-562), human acute monocytic leukemia cells (THP-1), and human lung carcinoma cells (A549) for their antiproliferative effects and against human cervix carcinoma cells (HeLa) for their cytotoxic effect. The antiproliperative and cytotoxic effects were tested via CellTiter-Blue and methylene blue assay as previously described [27]. In this assay, K-562 (DSM ACC 10), THP-1 (DSM ACC 16), and HeLa (DSM ACC 57) were maintained in Roswell Park Memorial Institute (RPMI) 1640 medium (Cambrex 12-167F) while HUVEC (ATCC CRL-1730) and A549 (DSM ACC 107) were cultured in Dulbecco’s Modified Eagle’s Medium (DMEM) (Cambrex 12-614F). Cells that were grown in the appropriate cell culture medium were supplemented with 10 mL/L ultraglutamine 1 (Cambrex 17-605E/U1), 550 µL/L (50 mg/mL) gentamicin sulfate (Cambrex 17-518Z), and 10% heat inactivated fetal bovine serum (GIBCO Life Technologies 10270-106) at 37 °C. The tested compounds were dissolved in DMSO, and the cells were seeded in 96-well plates at a density of 1 × 10^4^ cells/well. As for the antiproliferative effect of the compounds, the cells were incubated for 72 h, and GI_50_ values were evaluated to be defined as the concentration causing 50% inhibition of proliferation compared to the untreated control. With regard to the cytotoxic assay, HeLa cells were pre-incubated for 48 h without the test compounds. Then, the cells were exposed with different concentrations of compounds and incubated for 72 h. After that, the adherent HeLa cells were fixed by glutaraldehyde and stained with a 0.05% solutions of methylene blue (SERVA 29198) for 15 min. CC_50_ was evaluated to be defined as the concentration required for the death of 50% of the cell monolayer as compared to control groups. Under our experimental conditions, the optical density measured from the CellTiter-Blue reagent and methylene blue assay is proportional to the number of viable cells. In this experiment, absorbances were measured at 570 nm against the reference wavelength of 600 nm (CellTiter-Blue assay) and at 660 nm (methylene blue assay). Doxorubicin (Adriamycin^®^) and imatinib (Gleevec^®^) were used as positive controls for HUVEC, K-562, and HeLa cells. A repeat determination has been conducted in all experiments, and four replicates were assayed. The calculations of the different values of GI_50_ and CC_50_ were performed with software Magellan version 3.00 (Tecan Trading AG, Maennedorf, Switzerland).

## 4. Conclusions

The chemical investigation of the underground parts of *I. tenuifolia* afforded in the isolation of five unprecedented chromane derivatives (**1**–**3**, **9**, **10**) includes an unusual macrolide termed moniristenulide (**1**), together with seven known compounds (**4**–**8**, **11**–**12).** Notably, eight out of nine isolated flavonoids have a rare 2′,3′-disubstituted configuration on the B-ring, out of which the compounds bearing *ortho*-dihydroxyl groups in B-ring, namely **3**, **5,** and **10,** showed the broadest antimicrobial activity. On top of that, the molecules with methoxy or methylenedioxy substitution on the A-ring together with *ortho*-hydroxyl groups on the B-ring showed promising antiproliferative activities against leukemia cell lines in combination with low cytotoxicity, as shown for compounds **4**–**6** and **10**.

## Figures and Tables

**Table 1:** 1D and 2D NMR (500 MHz) data of moniristenulide (**1**) in DMSO-*d*_6_ (δ in ppm, *J* in Hz).

Position	δ_H_	δ_C_	HMBC	^1^H-^1^H COSY	ROESY
2	4.58 brd (13.4)	76.9	C-4, C-3	H-3_ax_, H-3_eq_, H-6′	H-3_eq_, H-3′, H-4_b_′, H-6′
3_ax_	3.21 m	38.5	C-4, C-6′	H-2, H-3_eq_	H-3_eq_, 6′-OH
3_eq_	2.40 m		C-4, C-10	H-2, H-3_ax_	H-2, H-6′, H-3_ax_
4	-	198.5	-	-	-
5	-	163.1	-	-	-
6	5.95 d (2.0)	97.3	C-7, C-8, C-10	-	-
7	-	164.1	-	-	-
8	6.14 d (2.0)	94.7	C-4, C-6, C-7, C-10	-	H-3′, H-1″
9	-	162.4	-	-	-
10	-	103.4	-	-	-
1′	-	164.8	-	-	-
2′	5.75 d (15.6)	120.2	C-1′, C-3′, C-4′	H-3′, H-4′_ab_	H-3′
3′	6.94 m	150.1	C-1′, C-2′, C-4′, C-5′	H-2′, H-4′_ab_	H-2, H-8, H-5′_b_
4′_a_	2.41 m	25.3	-	H-3′, H-4′_b_	-
4′_b_	2.07 m		C-2′, C-3′ C-5′, C-6′	H-3′, H-5′_b_, H-4′_a_	-
5′_a_	2.01 m	29.7	C-2, C-3′, C-4′	H-6′, H-4′_a_, H-5′_b_	-
5′_b_	1.81 m		C-3′, C-4′, C-6′	H-6′, H-5′_a_	-
6′	3.54 m	70.9	C-2	H-2, 6′-OH, H-5′_ab_	H-2, H-3_eq_, H-4′_b_, 6′-OH
1″	5.09 d (7.7)	98.0	C-7, C-5″	H-2″	H-8, H-3″, H-5″
2″	3.27 overlap	72.8	C-1″, C-3″, C-4″	H-1″, 2″-OH	-
3″	3.32 overlap	76.3	C-2″	H-4″, 3″-OH	-
4″	3.08 m	70.2	C-3″, C-6″	H-5″, 4″-OH	-
5″	3.71 t (10.4, 1.2)	74.4	C-1″, C-3″, C-4″, C-6″	H-4″, H-6″_a_, H-6″_b_	H-1″, H-3″, H-6″_a_
6″_a_	4.36 brd (11.5)	63.2	C-1′, C-1″, C-5″	H-5″, H-6″_b_	H-2, H-5″, H-6″_b_, 4″-OH
6″_b_	4.06 brd (10.9)		C-1′, C-5″	H-5″, H-6″_a_	-
5-OH	12.04 s	-	C-4, C-5, C-6, C-10	-	-
6′-OH	5.14 d (5.9)	-	C-2, C-5′, C-6′	H-6′	H-3_ax_
2″-OH	5.50 d (5.1)	-	C-1″, C-2″, C-3″	H-2″	H-2″
3″-OH	5.25 d (5.1)	-	C-2″, C-3″, C-4″	H-3″	-
4″-OH	5.36 d (5.1)	-	C-3″, C-4″, C-5″	H-4″	H-4″, H-6″_a_

**Table 2:** ^1^H NMR (500 MHz) and ^13^C NMR (125 MHz) data of compounds **2**, **3**, **9**, and **10** (δ in ppm, *J* in Hz).

Position	2 *		3 **		9 **		10 *	
	δ_H_	δ_C_	δ_H_	δ_C_	δ_H_	δ_C_	δ_H_	δ_C_
2	5.25 dd (2.6, 4.4)	67.5	5.70 dd (2.9, 12.9)	74.0	8.44 s	157.1	8.16 s	156.3
3	2.27 dt (2.8, 13.8)	26.5	3.17 dd (12.9, 17.0)	41.1	-	118.9	-	122.8
	2.15 dt (2.8, 13.8)		2.69 dd (3.0, 17.0)		-		-	
4	5.64 dd (2.8, 4.4)	62.4	-	196.4	-	180.5	-	182.4
5	-	141.1	-	163.5	-	156.8	-	158.1
6	-	129.9	5.88 d (2.0)	95.8	-	128.3	-	128.9
7	-	150.5	-	166.6	-	158.3	-	159.7
8	6.08 s	92.5	5.90 d (2.0)	94.9	6.64 s	96.1	6.54 s	96.9
9	-	148.9	-	163.2	-	149.2	-	149.7
10	-	106.1	-	101.7	-	104.8	-	104.9
1′	-	121.3	-	125.5	-	120.0	-	119.8
2′	-	153.6	-	142.6	-	155.1	-	147.7
3′	6.87 overlap	117.2	-	145.2	7.27 d (8.2)	115.5	-	142.6
4′	7.20 ddd (1.7, 7.4, 7.4)	130.6	6.79 dd (1.4, 7.9)	115.2	7.37 overlap	129.7	7.03 dd (1.3, 7.9)	115.6
5′	6.89 overlap	120.4	6.69 t (7.8)	119.1	7.09 t (7.5)	121.5	6.92 t (7.9)	122.2
6′	7.33 dd (1.7, 7.5)	130.9	6.88 dd (1.4, 7.8)	117.1	7.35 overlap	131.9	6.71 dd (1.5, 7.8)	120.3
5-OCH_3_	4.09 s (3H)	60.1	-	-	-	-	-	-
-OCH_2_O-	5.76 d (1.5)	100.8	-	-	-	-	-	-
	5.81 d (1.5)							
5-OH	-	-	12.12 s	-	12.68 s	-	12.07 s	-
7-OH	-	-	10.79 s	-	-	-		
2′-OH	-	-	8.71 s	-	-	-	8.48 s	-
3′-OH	-	-	9.51 s	-	-	-	6.09 s	-
1″	-	-	-	-	4.89 d (7.8)	101.1	-	-
2″	-	-	-	-	3.14 m	73.3	-	-
3″	-	-	-	-	3.25 m	76.5	-	-
4″	-	-	-	-	3.12 m	69.7	-	-
5″	-	-	-		3.32 m	77.1	-	-
6a″	-	-	-	-	3.70 dd (5.0, 11.5)	60.7	-	-
6b″	-	-	-	-	3.46 m		-	
2″-OH	-	-	-	-	5.05 d (5.0)	-	-	-
3″-OH	-	-	-	-	5.01 overlap	-	-	-
4″-OH	-	-	-	-	5.02 overlap	-	-	-
6″-OH	-	-	-	-	4.57 t (5.8, 11.5)	-	-	-
6-OCH_3_	-	-	-	-	3.79 s (3H)	61.0	3.91 s (3H)	61.9
7-OCH_3_	-	-	-	-	3.94 s (3H)	56.6	3.99 s (3H)	56.7

*—in CDCl_3_, **—in DMSO-*d*_6._

**Table 3:** Antimicrobial activity using agar diffusion method of some isolated compounds from *I. tenuifolia*.

Test Microorganism	Inhibition Zone of Test Microorganism (mm)						
	1	2	3	5	9	10	Standard
	Gram-positive						Ciprofloxacin
*Staphylococcus aureus* (511 B3)	-	-	-	15	-	-	19
*Bacillussubtilis* (6633 B1)	-	12	12	13	-	13	29
*Staphylococcus aureus* (MRSA 134/94 R9)	-	-	-	13	-	-	-
*Enterococcus faecalis* (VRE 1528 R10)	13	14	17	13	13	13	16
	Gram-negative						
*Escherichiacoli* (458 B4)	-	-	-	-	-	-	32
*Pseudomonas aeruginosa* (SG137 B7)	-	-	-	-	-	-	25
*Pseudomonas aeruginosa* (K799/61 B9)	-	-	-	17	-	-	35
*Mycobacterium vaccae* (10670 M4)	-	17	23	20	-	16	22
	Fungi						Amphotericin B
*Sporobolomyces salmonicolor* (549 H4)	-	-	-	-	-	-	19
*Candida albicans* (C.alb.H8)	-	-	-	13	-	13	20
*Penicillium notatum* (JP36 P1)	-	-	-	16	-	-	19

VRE—Vancomycin resistant *Enterococci*; MRSA—Methicillin resistant *Staphylococcus aureus*;—showed no activity.

**Table 4:** Antiproliferative and cytotoxic activity of the plant extract and isolated compounds from *I. tenuifolia*.

Compound	Antiproliferative Effect, GI_50_ (µM) (CI 95%)				Cytotoxicity CC_50_ (µM) (CI 95%)
	HUVEC	K-562	THP-1	A549	HeLa
**1**	>100	>100	>100	>100	>100
**2**	35.2 (35.0–35.4)	31.5 (31.2–31.8)	29.2 (29.1–29.3)	>100	42.6 (41.8–43.4)
**3**	>100	32.3 (32.1–32.5)	>100	>100	>100
**4**	73.3 (72.8–73.8)	48.7 (46.8–50.6)	16.0 (15.9–16.1)	81.0 (80.7–81.3)	>100
**5**	69.0 (68.5–69.5)	44.3 (41.6–47.0)	16.5 (16.4–16.6)	>100	>100
**6**	68.2 (67.6–68.8)	56.0 (54.3–57.7)	16.9 (16.8–17.0)	70.9 (69.7–72.1)	>100
**7**	>100	97.6 (95.8–99.4)	>100	>100	>100
**8**	>100	>100	>100	>100	>100
**9**	>100	>100	>100	>100	>100
**10**	35.8 (35.4–36.2)	7.6 (7.5–7.7)	9.1 (9.07–9.13)	98.8 (97.7–99.9)	>100
**11**	>100	71.5 (71.1–71.9)	50.6 (49.0–52.2)	67.0 (66.6–67.4)	>100
**12**	>100	>100	>100	>100	>100
Imatinib	22.1 (20.9–23.2)	0.2 (0.20–0.21)	n.d	n.d	78.6 (77.3–80.0)
Doxorubicin	0.13 (0.10–0.16)	0.13 (0.12–0.14)	n.d	n.d	0.48 (0.46–0.49)
Plant extract (µg/mL)	≥50	47.2 (±1.8)	31.6 (±6.3)	>50	40.0 (±4.9)

The GI_50_ and CC_50_ values with 95% confidence intervals (CI 95%): 1–10 (very strong); 11–20 (strong); 21–50 (moderate); 51–100 (weak), and >100 (ineffective); n.d—not determined.

## Supplementary Materials

The following are available online, Figures S1–S10: 1D-, 2D-NMR, HR-ESI-MS, UV, CD, and HR-MS spectra of compound **1**, Figures S11–S17: 1D-, 2D-NMR, HR-ESI-MS, and UV spectra of compound **2**, Figures S18–S25: 1D-, 2D-NMR, HR-ESI-MS, UV, and CD spectra of compound **3**, Figures S26–S32: 1D-, 2D-NMR, HR-ESI-MS, and UV spectra of compound **9**, Figures S33–S39: 1D-, 2D-NMR, HR-ESI-MS, and UV spectra of compound **10**.

## Sample Availability

Samples of the compounds **3**–**12** are available from the authors.
