# Supplementary material for: Chromane Derivatives from Underground Parts of Iris tenuifolia and Their In Vitro Antimicrobial, Cytotoxicity and Antiproliferative Evaluation
Source: Molecules. 2021 Nov 5;26(21):6705. doi: 10.3390/molecules26216705 (PMC8588511; doi:10.3390/molecules26216705)
Supplement: Supplementary file 1 [file molecules-26-06705-s001.zip › molecules-1442043-supplementary materials.pdf]

molecules

Supplementary Materials:

Chromane Derivatives from Underground Parts of *Iris tenuifolia* and Their In Vitro Antimicrobial, Cytotoxicity and Antiproliferative Evaluation

Oldokh Otgon [1], Suvd Nadmid [2], Christian Paetz [3], Hans-Martin Dahse [4], Kerstin Voigt [4], Stefan Bartram [3], Wilhelm Boland [3] and Enkhmaa Dagvadorj [1,*]

[1] Department of Chemistry, School of Biomedicine, Mongolian National University of Medical Sciences, S.Zorig street 3, Ulaanbaatar 14210, Mongolia; oldokh.o@mnums.edu.mn (O.O.)
[2] Department of Pharmaceutical Chemistry, School of Pharmacy, Mongolian National University of Medical Sciences, S.Zorig street 3, Ulaanbaatar 14210, Mongolia; suvd.n@mnums.edu.mn (S.N.)
[3] Max Planck Institute for Chemical Ecology, Hans-Knoell-Strasse 8, D-07745 Jena, Germany; cpaetz@ice.mpg.de (C.P.); bartram@ice.mpg.de (S.B.); boland@ice.mpg.de (W.B.)
[4] Leibniz Institute for Natural Product Research and Infection Biology, Hans Knöll Institute (HKI), Adolf-Reichwein-Strasse 23, D-07745 Jena, Germany; hans-martin.dahse@leibniz-hki.de (H.-M.D.); kerstin.voigt@hki-jena.de (K.V.)
* Correspondence: enkhmaa.d@mnums.edu.mn (E.D.); Tel.: +976-99084787

Contents Lists Page

Figure S1 ^{1}H NMR spectrum of compound **1** (500 MHz, DMSO-d_6) 2
Figure S2 ^{13}C NMR spectrum of compound **1** (125 MHz, DMSO-d_6) 2
Figure S3 ^{1}H-^{1}H COSY spectrum of compound **1** (DMSO-d_6) 3
Figure S4 HSQC spectrum of compound **1** (DMSO-d_6) 3
Figure S5 HMBC spectrum of compound **1** (DMSO-d_6) 4
Figure S6 ROESY spectrum of compound **1** (DMSO-d_6) 4
Figure S7 HR-ESI-MS spectrum of compound **1** 4
Figure S8 UV spectrum of compound **1** 5
Figure S9 CD spectrum of compound **1** 5
Figure S10 HR-MS/MS fragmentation of compound **1** 5
Figure S11 ^{1}H NMR spectrum of compound **2** (500 MHz, $CDCl_3$) 6
Figure S12 ^{13}C NMR spectrum of compound **2** (125 MHz, $CDCl_3$) 6
Figure S13 ^{1}H-^{1}H COSY spectrum of compound **2** ($CDCl_3$) 7
Figure S14 HSQC spectrum of compound **2** ($CDCl_3$) 7
Figure S15 HMBC spectrum of compound **2** ($CDCl_3$) 7
Figure S16 HR-ESI-MS spectrum of compound **2** 8
Figure S17 UV spectrum of compound **2** 8
Figure S18 ^{1}H NMR spectrum of compound **3** (500 MHz, DMSO-d_6) 9
Figure S19 ^{13}C NMR spectrum of compound **3** (125 MHz, DMSO-d_6) 9
Figure S20 ^{1}H-^{1}H COSY spectrum of compound **3** (DMSO-d_6) 10
Figure S21 HSQC spectrum of compound **3** (DMSO-d_6) 10
Figure S22 HMBC spectrum of compound **3** (DMSO-d_6) 10
Figure S23 HR-ESI-MS spectrum of compound **3** 11
Figure S24 UV spectrum of compound **3** 11
Figure S25 CD spectrum of compound **3** 11
Figure S26 ^{1}H NMR spectrum of compound **9** (500 MHz, DMSO-d_6) 12
Figure S27 ^{13}C NMR spectrum of compound **9** (125 MHz, DMSO-d_6) 12

Figure S28 ^{1}H-^{1}H COSY spectrum of compound **9** (DMSO-d_6) 13
Figure S29 HSQC spectrum of compound **9** (DMSO-d_6) 13
Figure S30 HMBC spectrum of compound **9** (DMSO-d_6) 13
Figure S31 HR-ESI-MS spectrum of compound **9** 14
Figure S32 UV spectrum of compound **9** 14
Figure S33 ^{1}H NMR spectrum of compound **10** (500 MHz, $CDCl_3$) 15
Figure S34 ^{13}C NMR spectrum of compound **10** (125 MHz, $CDCl_3$) 15
Figure S35 ^{1}H-^{1}H COSY spectrum of compound **10** ($CDCl_3$) 16
Figure S36 HSQC spectrum of compound **10** ($CDCl_3$) 16
Figure S37 HMBC spectrum of compound **10** ($CDCl_3$) 16
Figure S38 HR-ESI-MS spectrum of compound **10** 17
Figure S39 UV spectrum of compound **10** 17

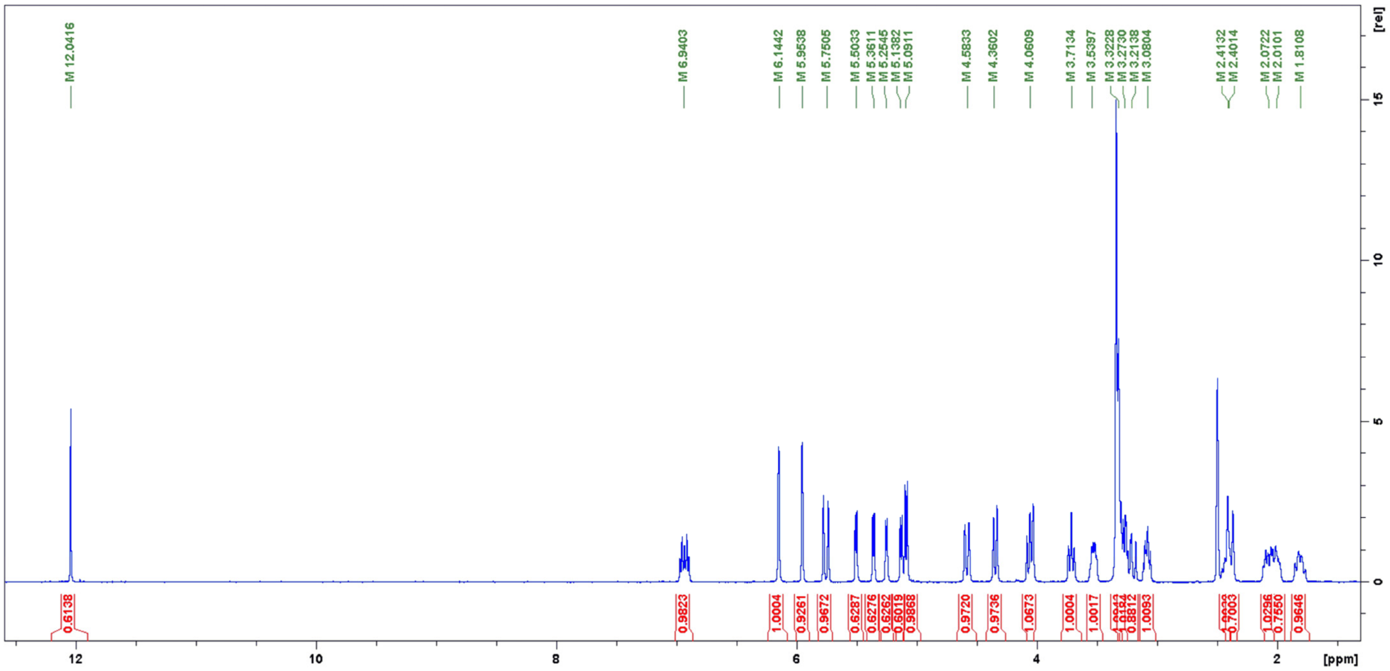

Figure S1 ^{1}H NMR spectrum of compound **1** (500 MHz, DMSO-d_6)

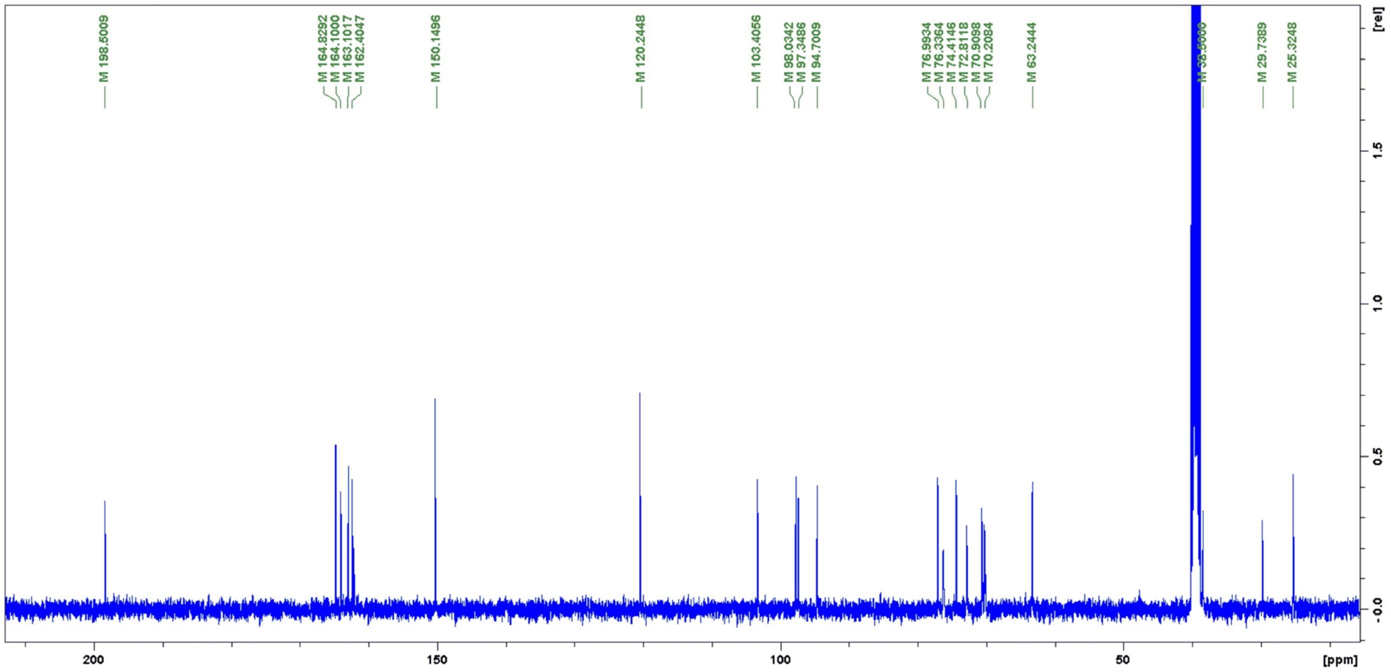

Figure S2 ^{13}C NMR spectrum of compound **1** (125 MHz, DMSO-d_6)

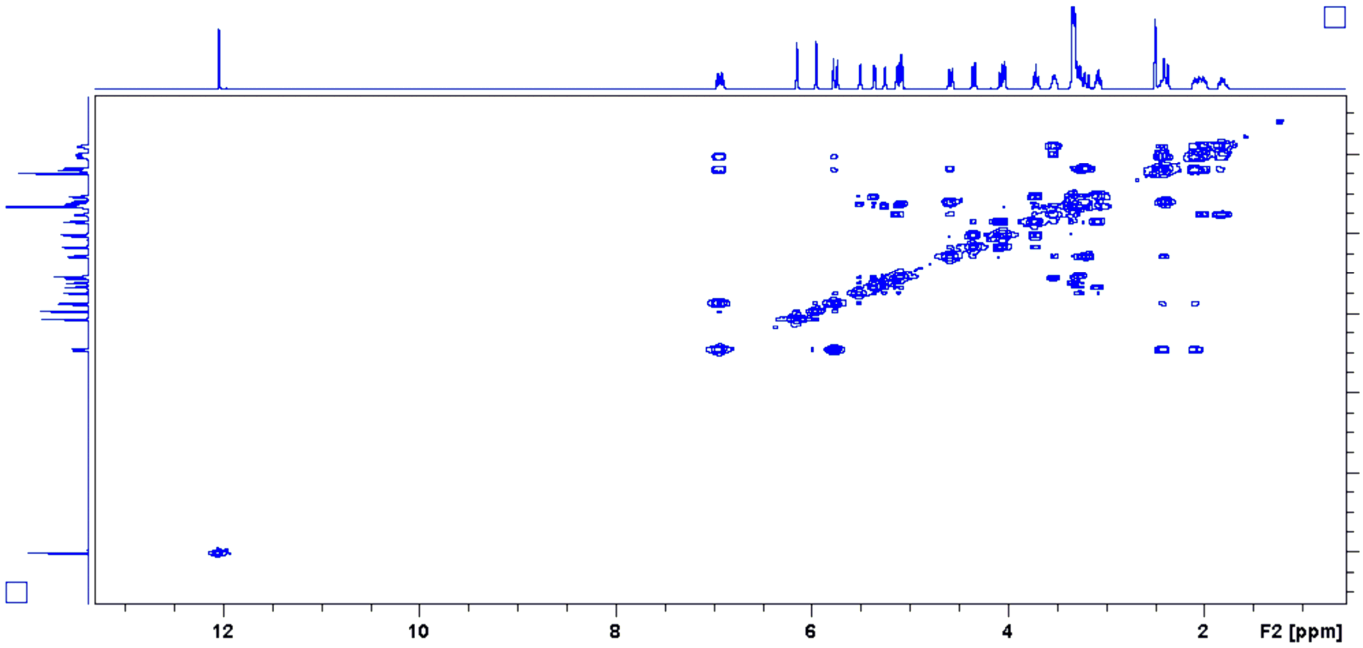

Figure S3 ^{1}H-^{1}H COSY spectrum of compound **1** (DMSO-d_6)

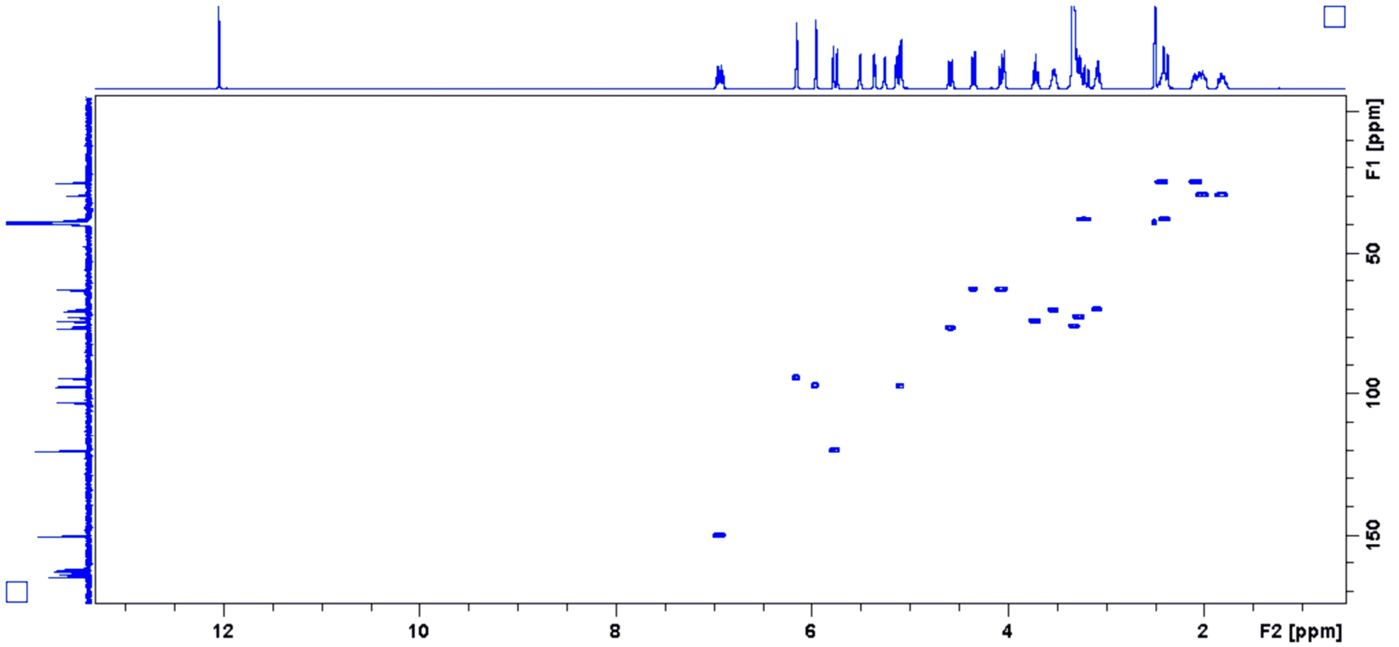

Figure S4 HSQC spectrum of compound **1** (DMSO-d_6)

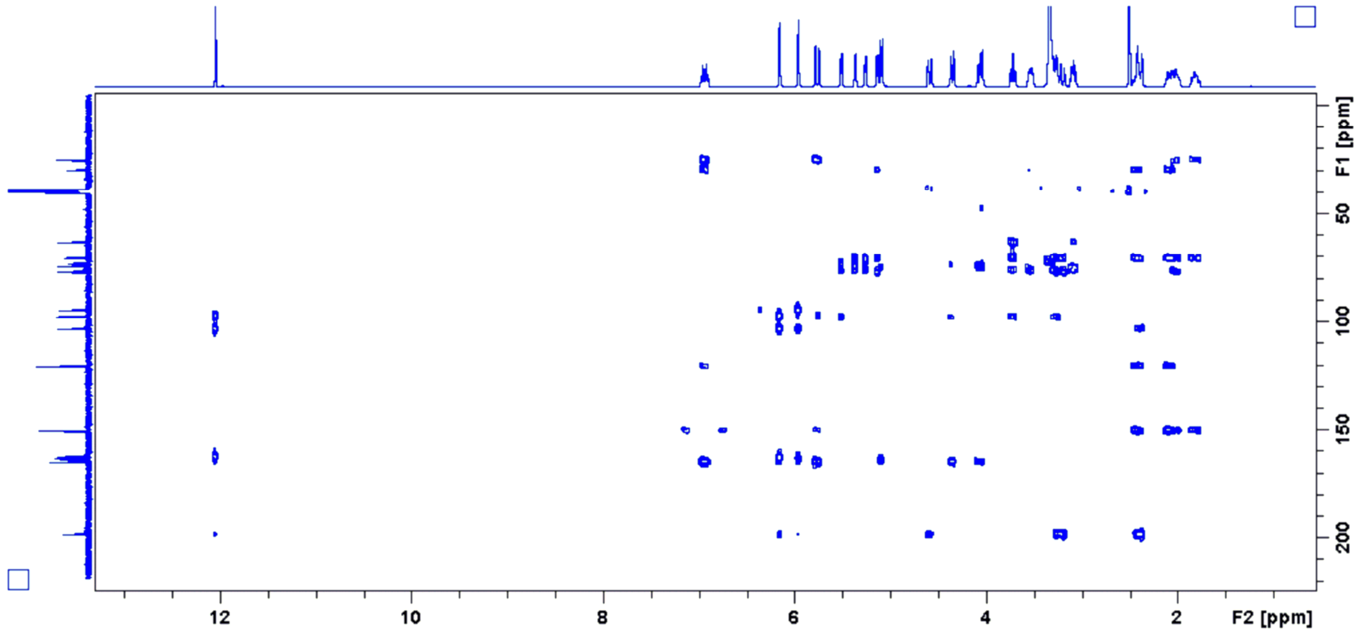

Figure S5 HMBC spectrum of compound **1** (DMSO-d_6)

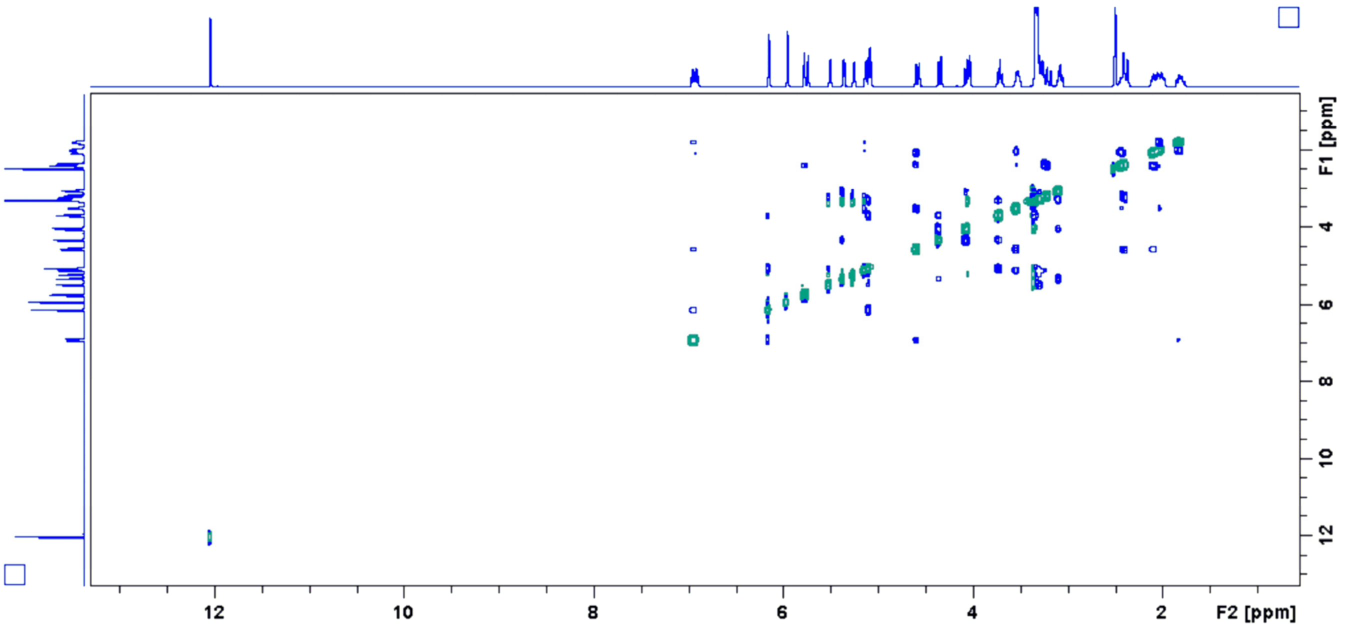

Figure S6 ROESY spectrum of compound **1** (DMSO-d_6)

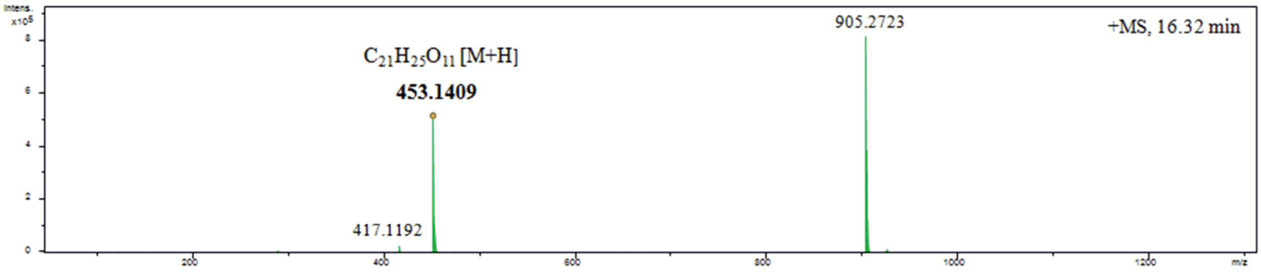

Figure S7 HR-ESI-MS spectrum of compound **1**

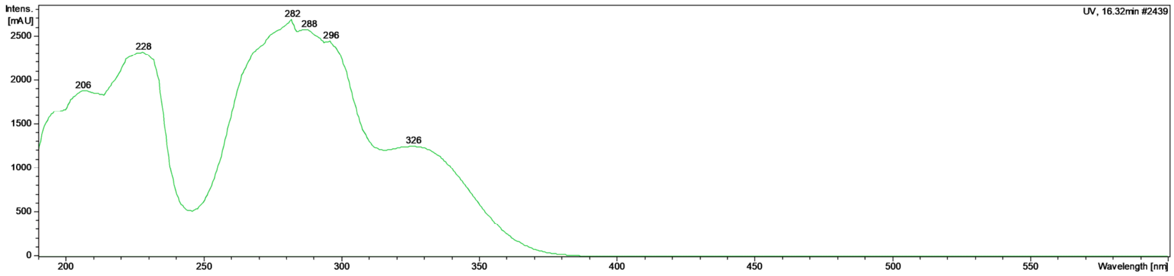

Figure S8 UV spectrum of compound **1**

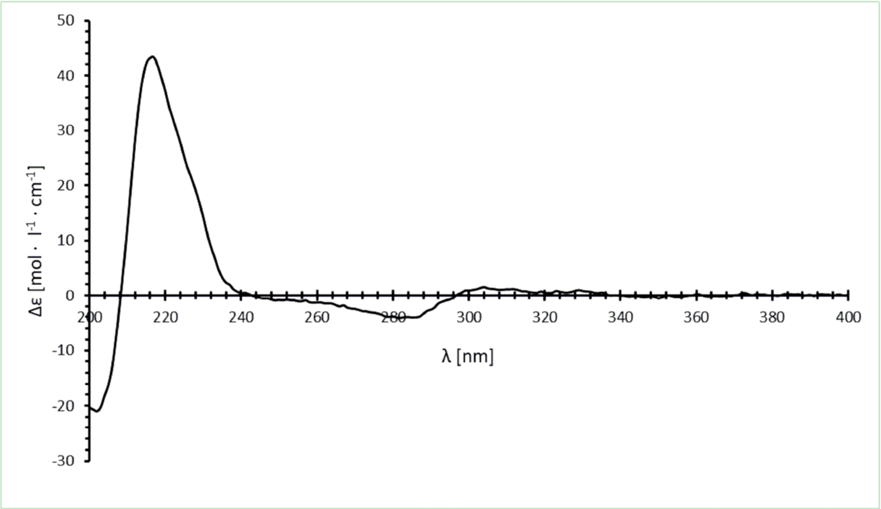

Figure S9 CD spectrum of compound **1**

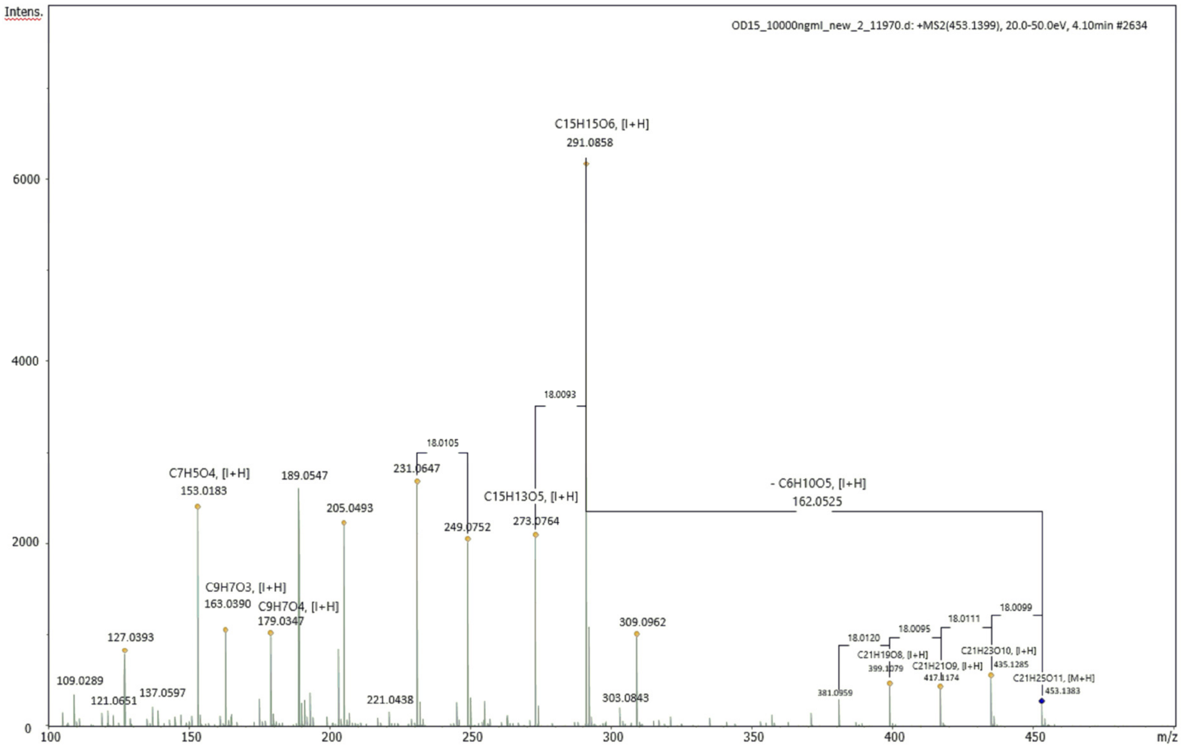

Figure S10 HR-MS/MS fragmentation of compound **1**

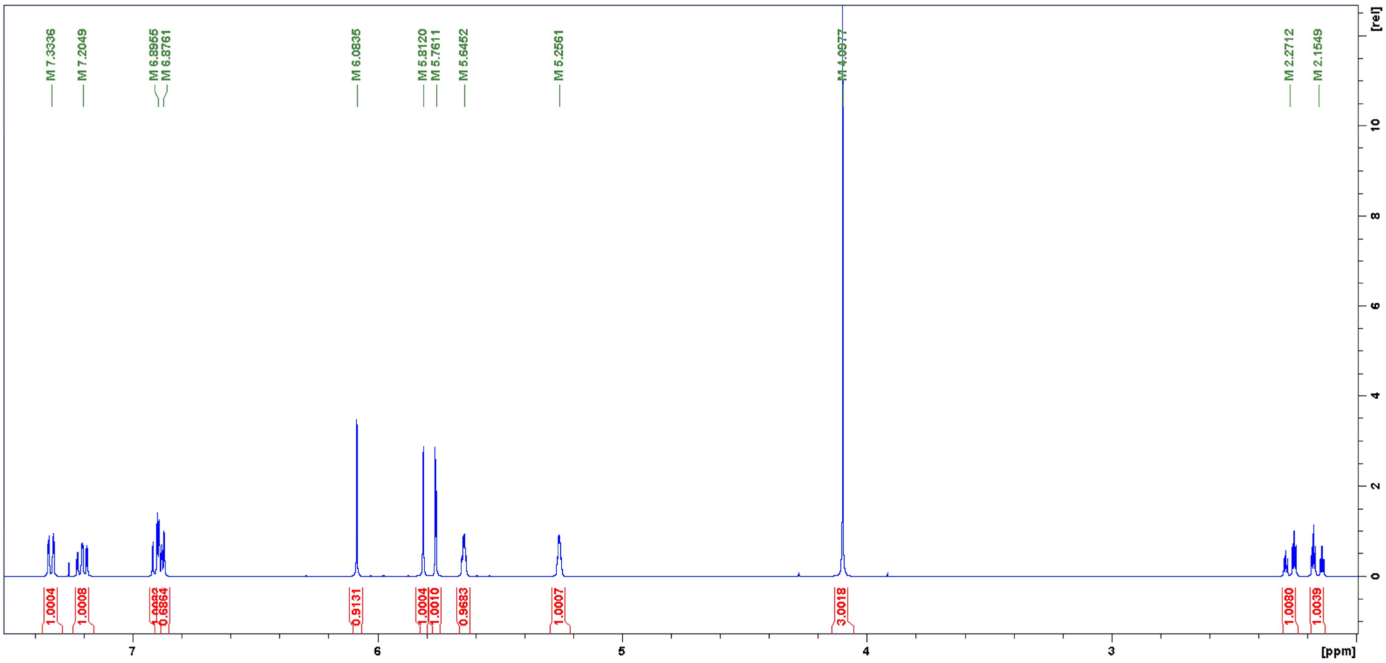

Figure S11 ^{1}H NMR spectrum of compound **2** (500 MHz, $CDCl_3$)

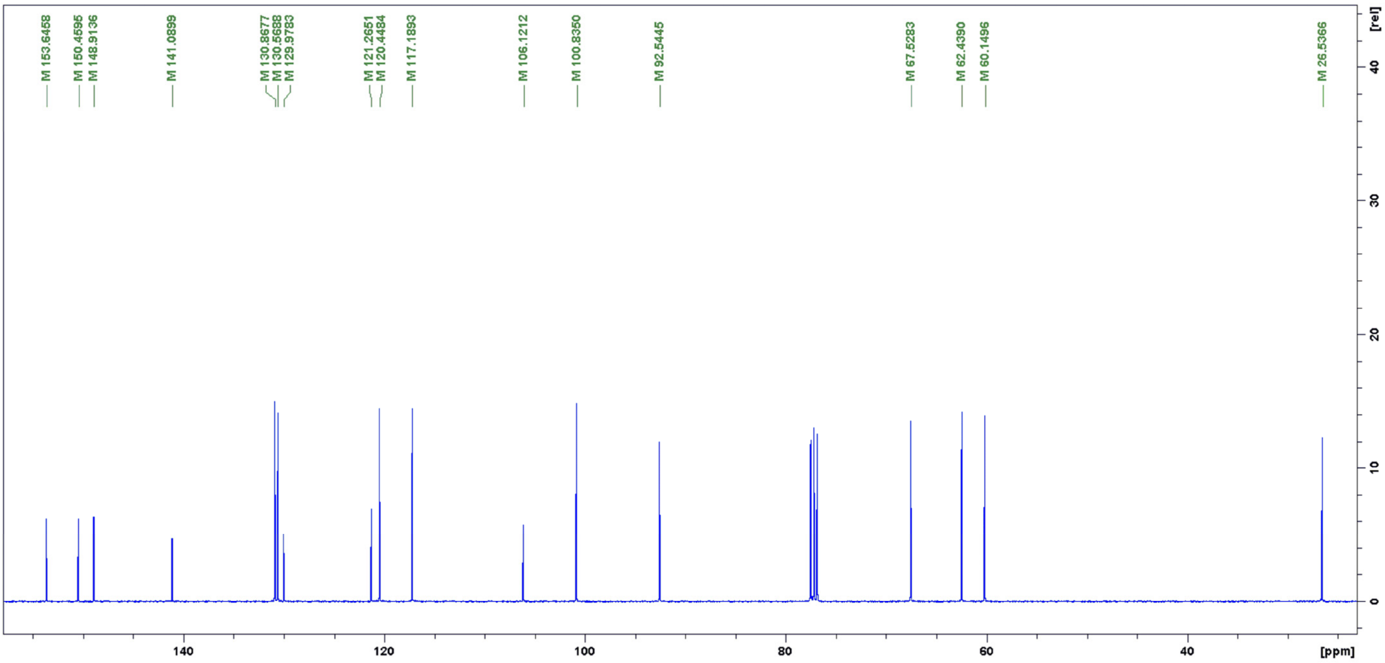

Figure S12 ^{13}C NMR spectrum of compound **2** (125 MHz, $CDCl_3$)

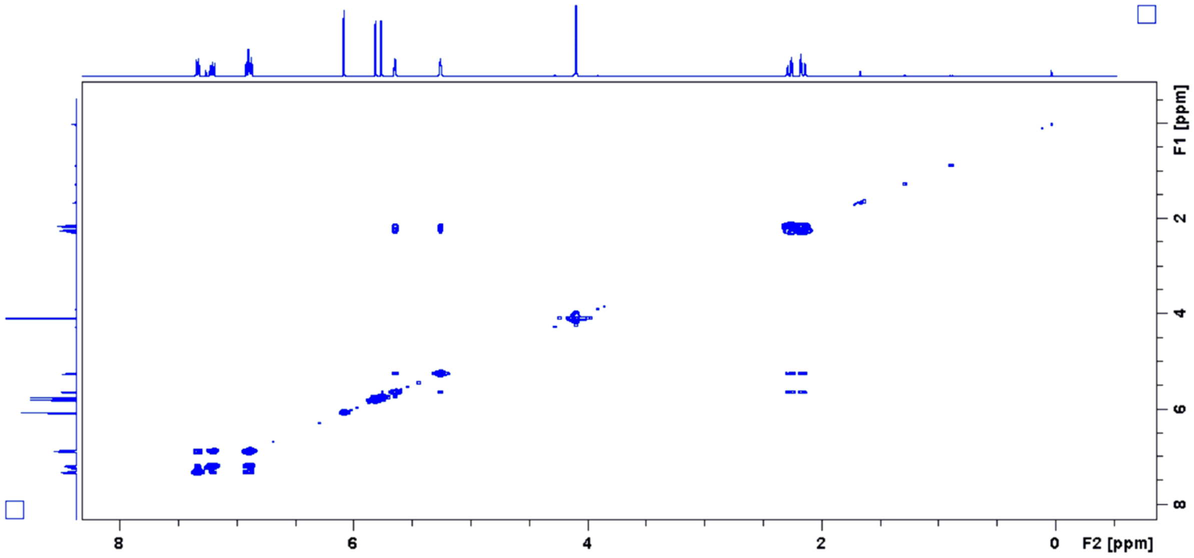

Figure S13 ^{1}H-^{1}H COSY spectrum of compound **2** ($CDCl_3$)

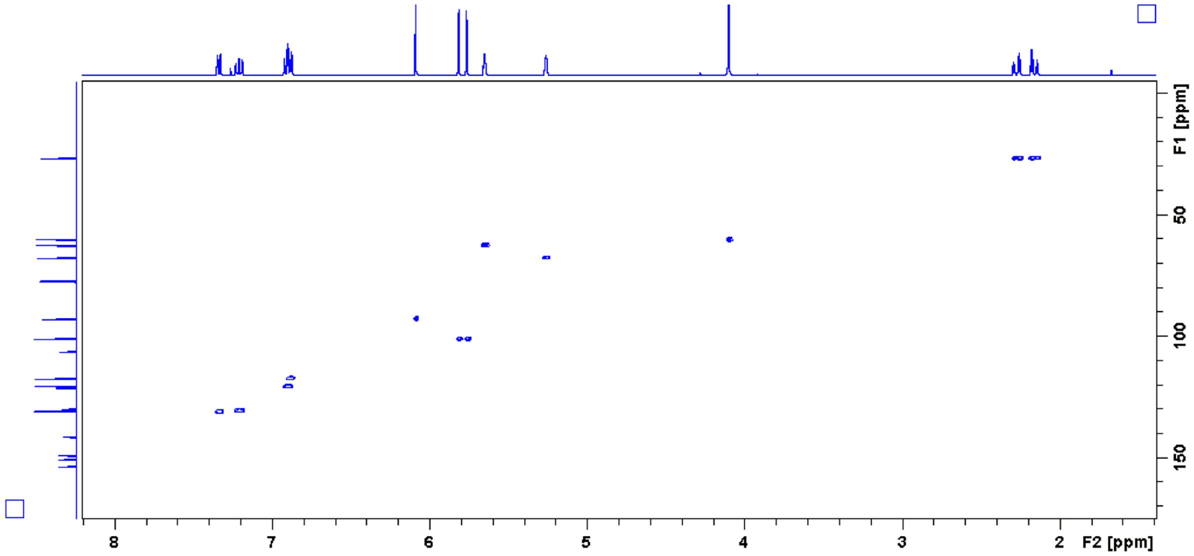

Figure S14 HSQC spectrum of compound **2** ($CDCl_3$)

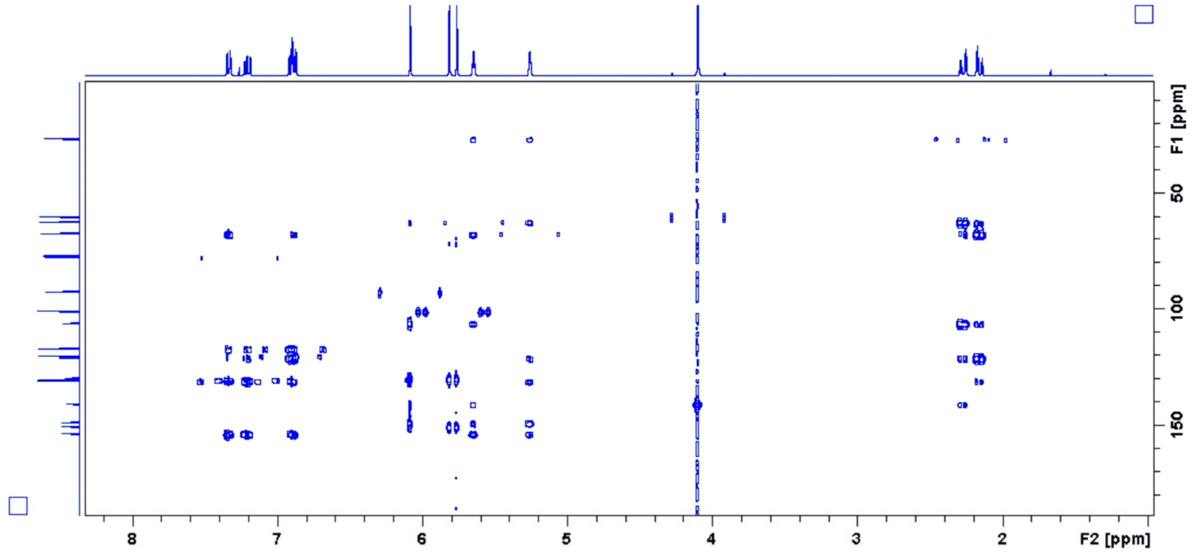

Figure S15 HMBC spectrum of compound **2** ($CDCl_3$)

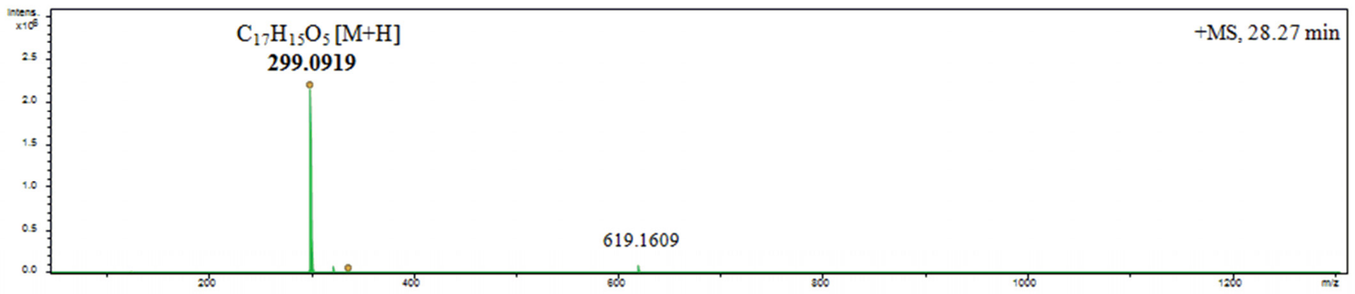

Figure S16 HR-ESI-MS spectrum of compound **2**

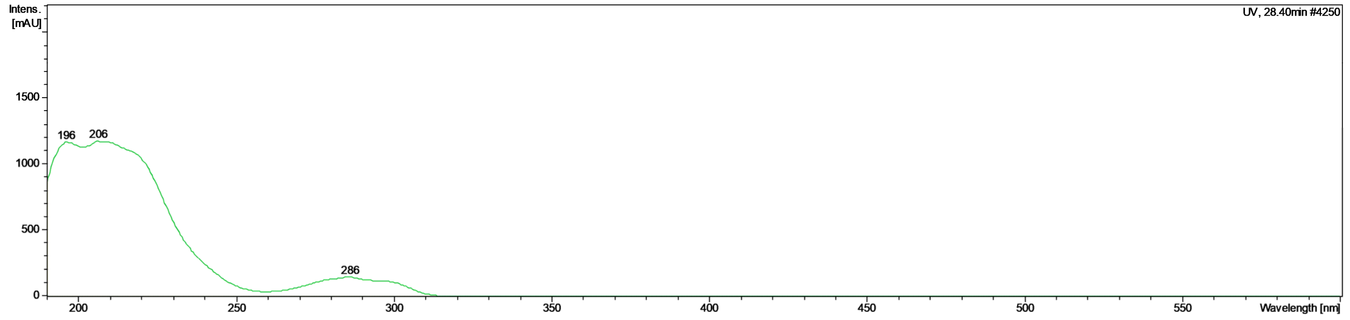

Figure S17 UV spectrum of compound **2**

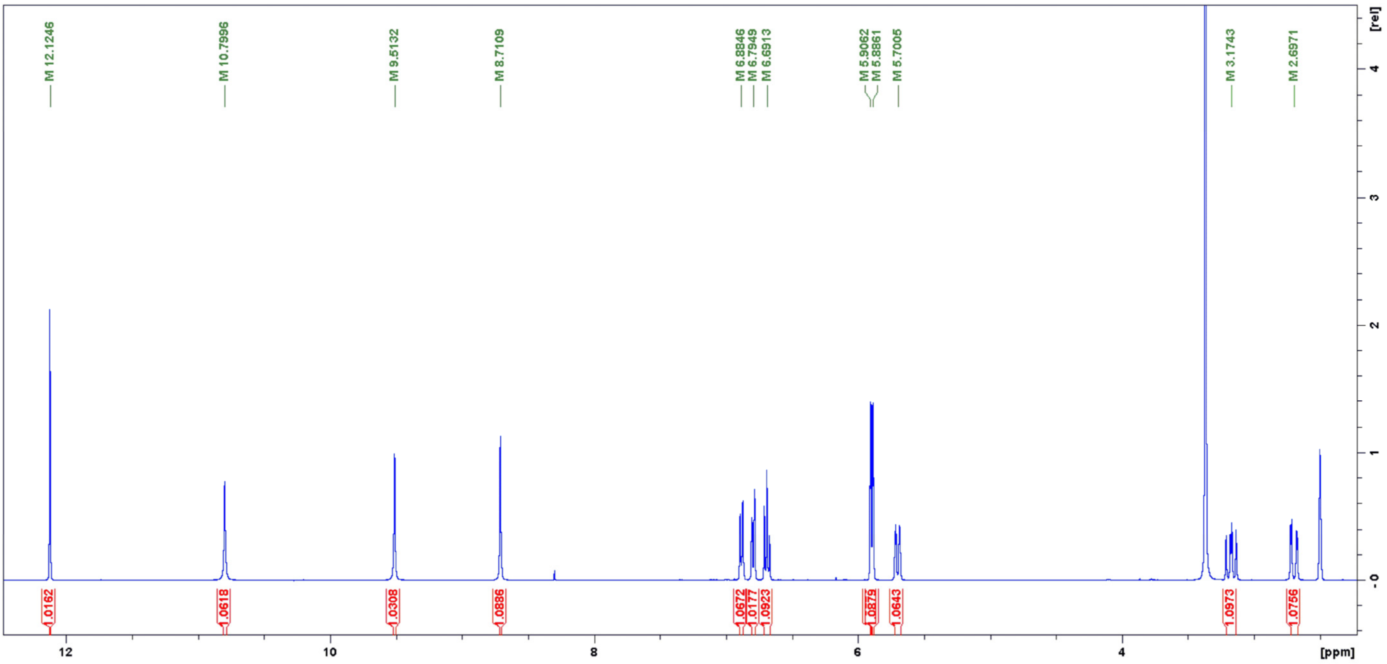

Figure S18 ^{1}H NMR spectrum of compound **3** (500 MHz, DMSO-d_6)

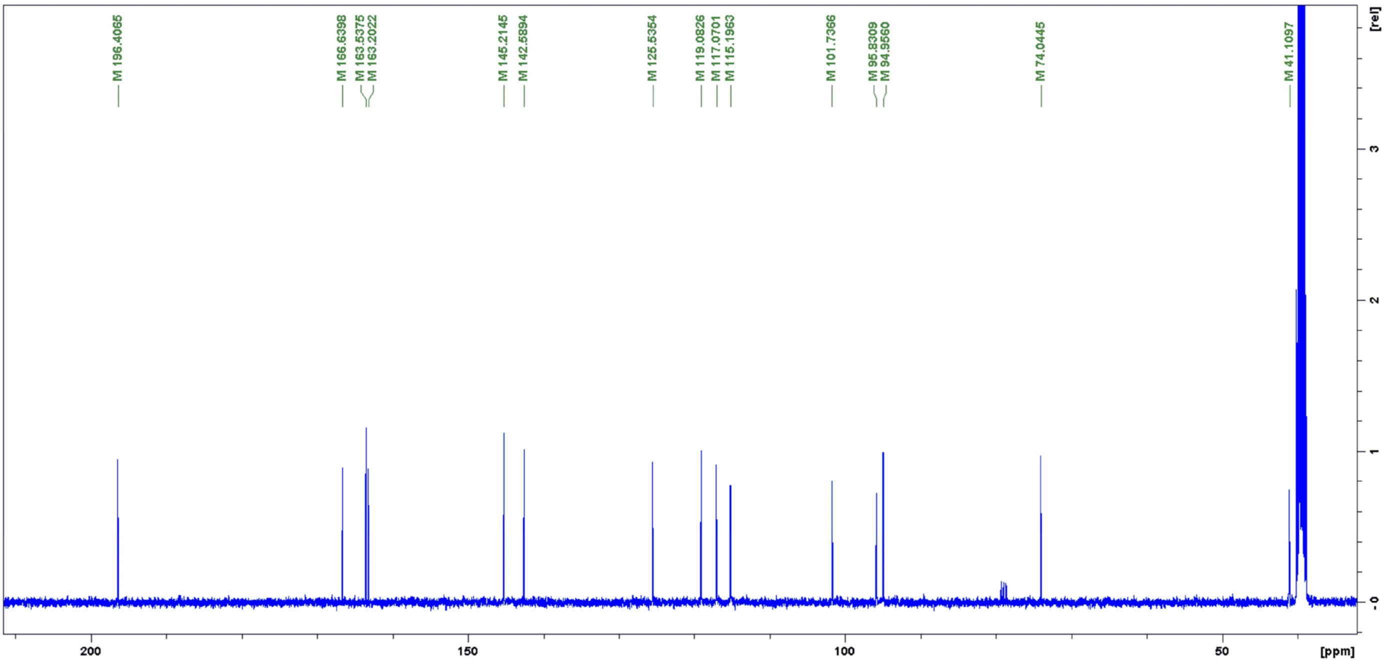

Figure S19 ^{13}C NMR spectrum of compound **3** (125 MHz, DMSO-d_6)

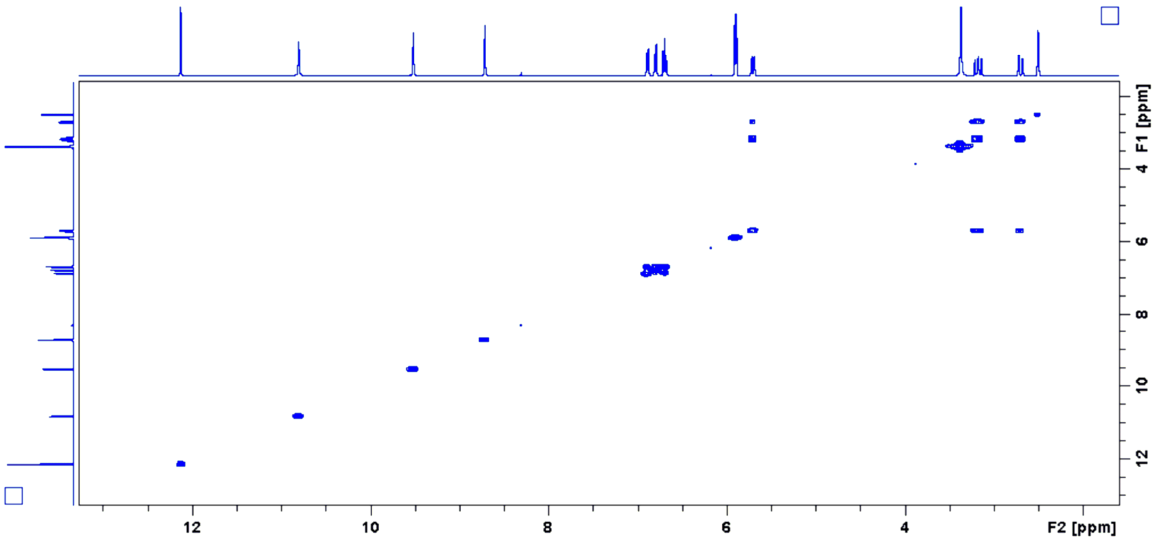

Figure S20 ^{1}H-^{1}H COSY spectrum of compound **3** (DMSO-d_6)

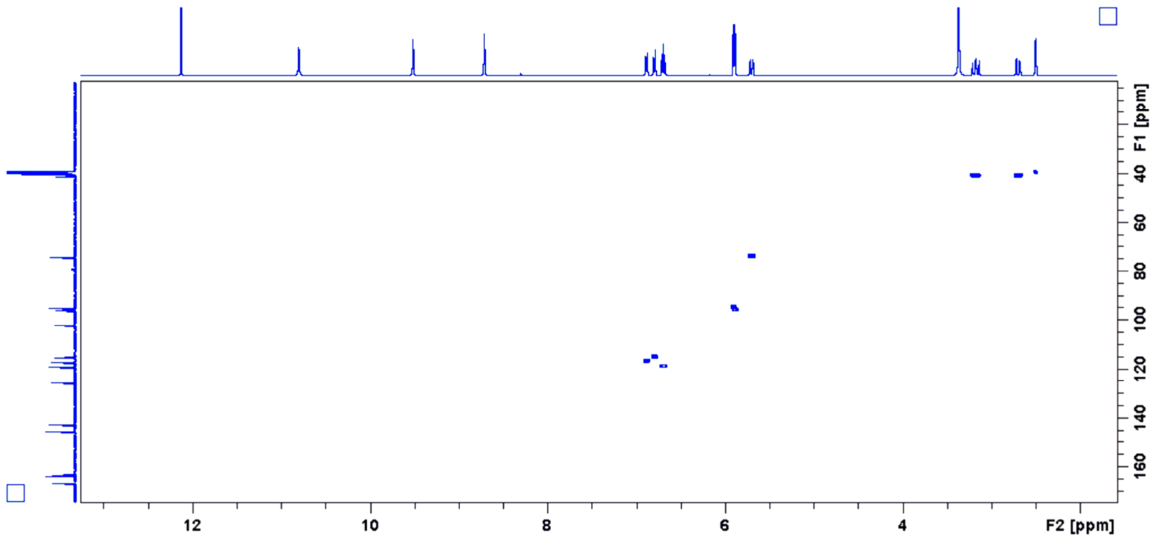

Figure S21 HSQC spectrum of compound **3** (DMSO-d_6)

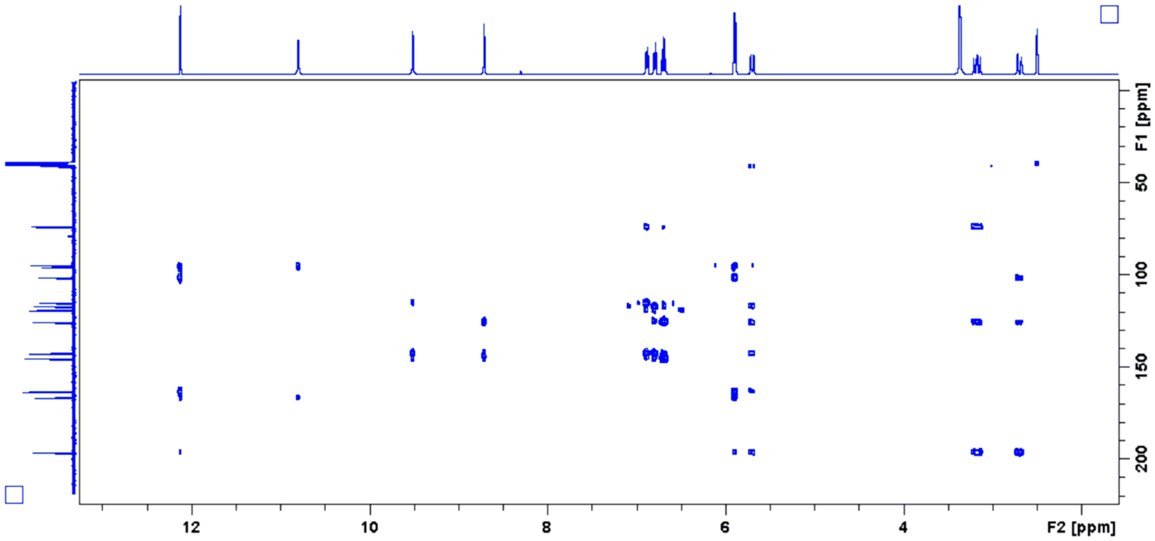

Figure S22 HMBC spectrum of compound **3** (DMSO-d_6)

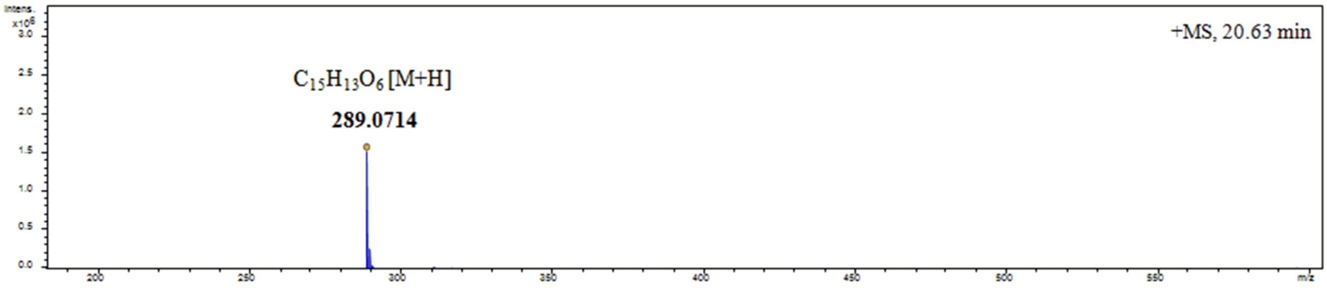

Figure S23 HR-ESI-MS spectrum of compound **3**

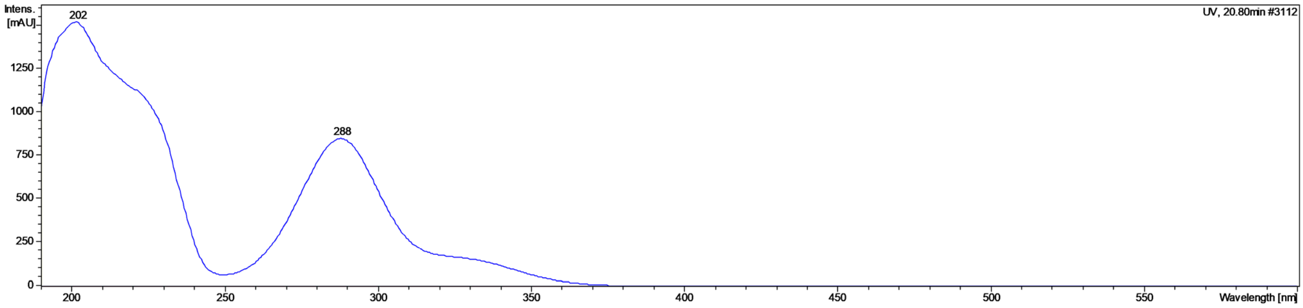

Figure S24 UV spectrum of compound **3**

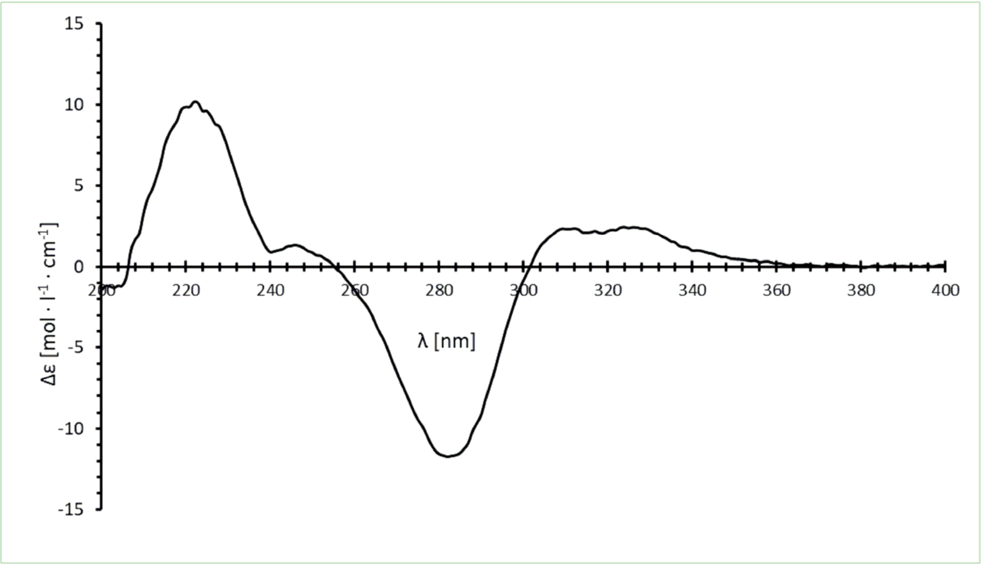

Figure S25 CD spectrum of compound **3**

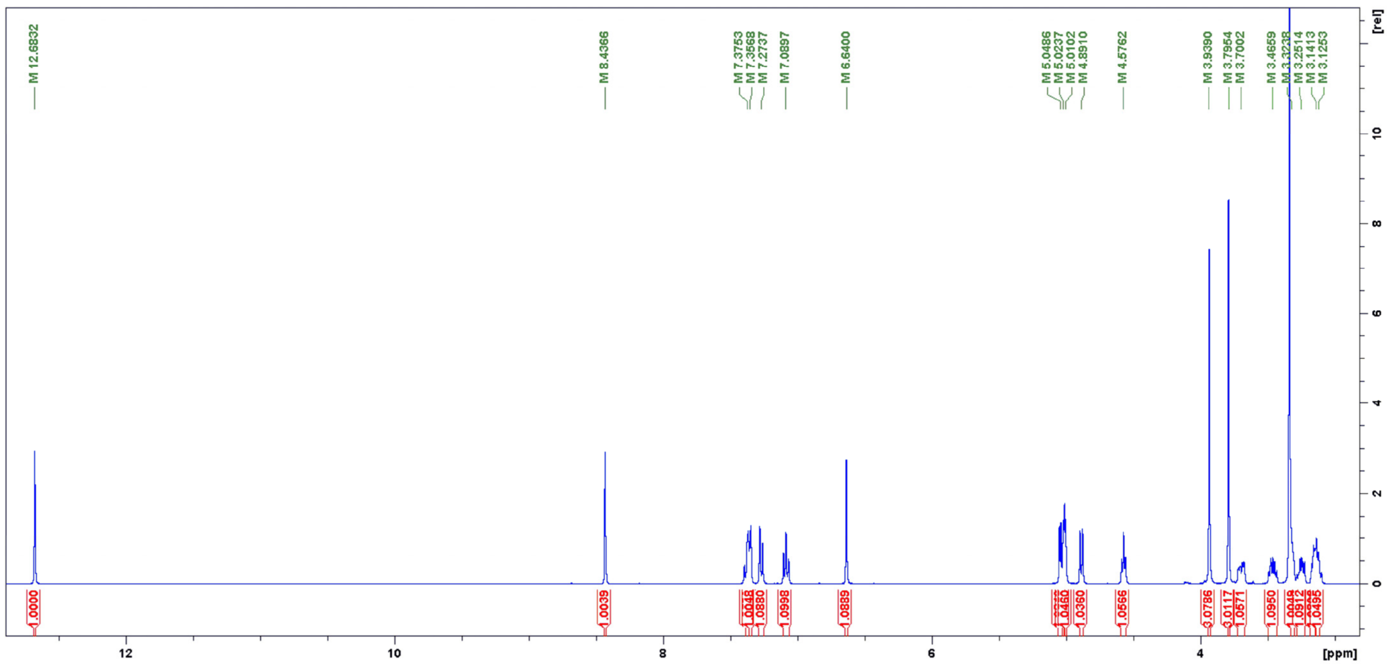

Figure S26 ¹H NMR spectrum of compound **9** (500 MHz, DMSO-d_6)

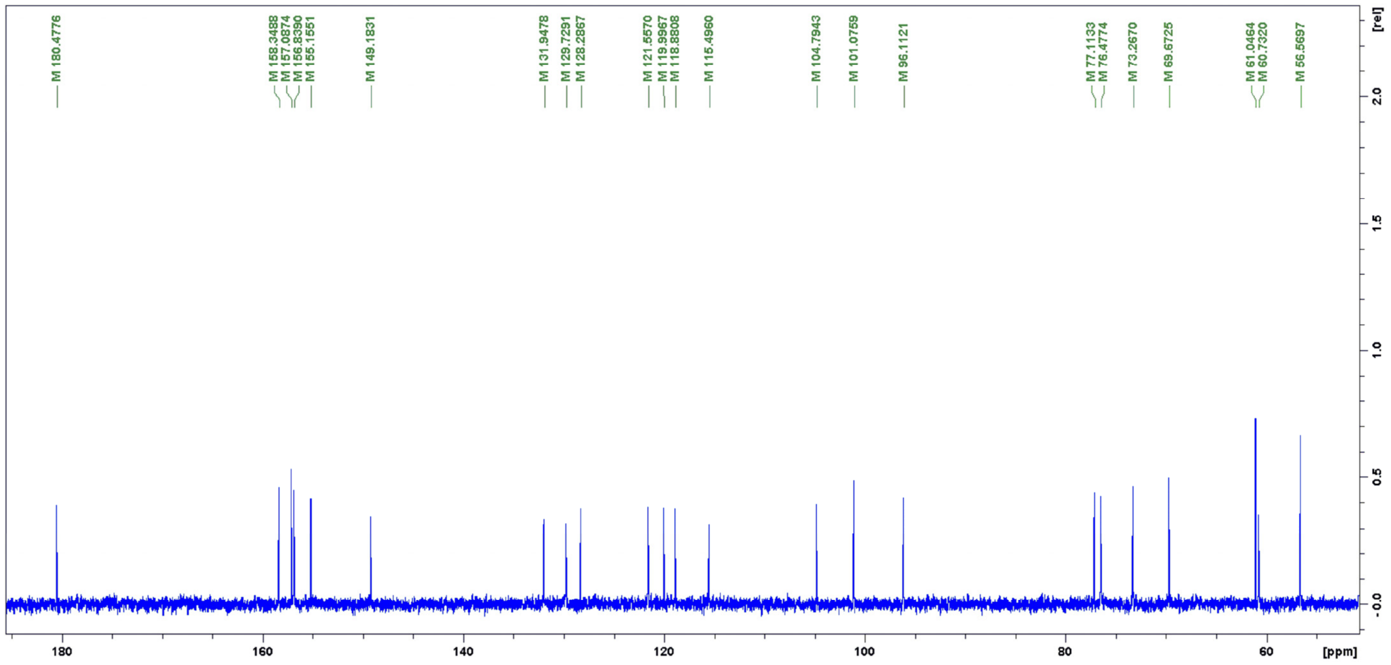

Figure S27 ¹³C NMR spectrum of compound **9** (125 MHz, DMSO-d_6)

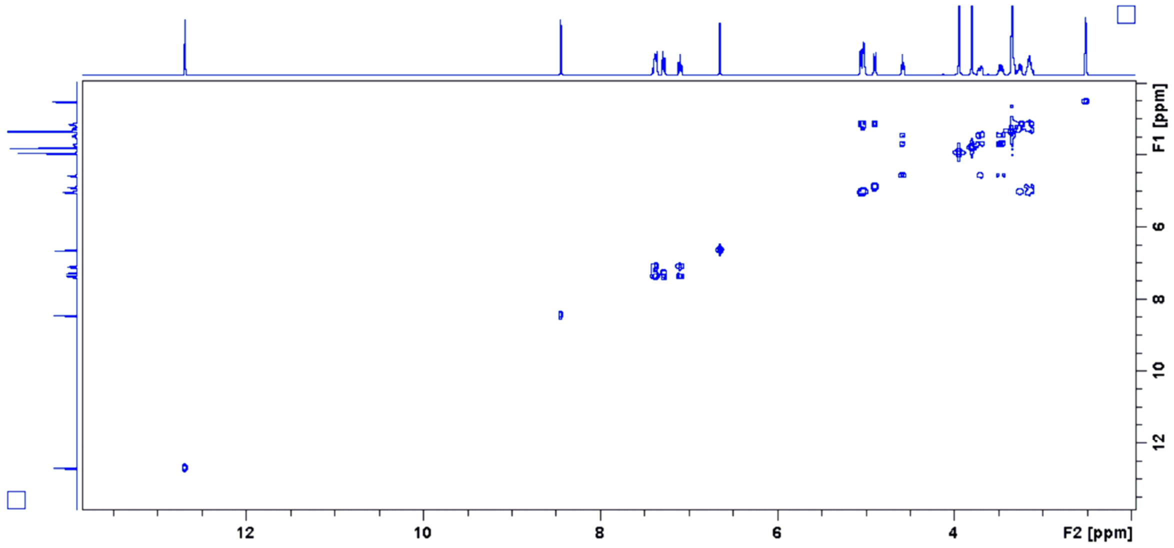

Figure S28 ^{1}H-^{1}H COSY spectrum of compound **9** (DMSO-d_6)

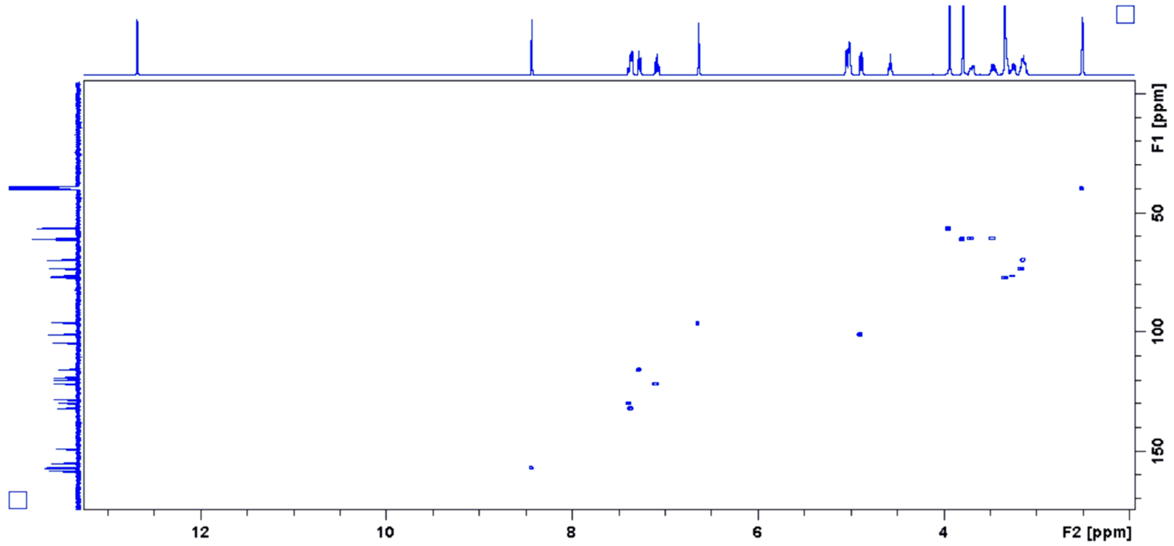

Figure S29 HSQC spectrum of compound **9** (DMSO-d_6)

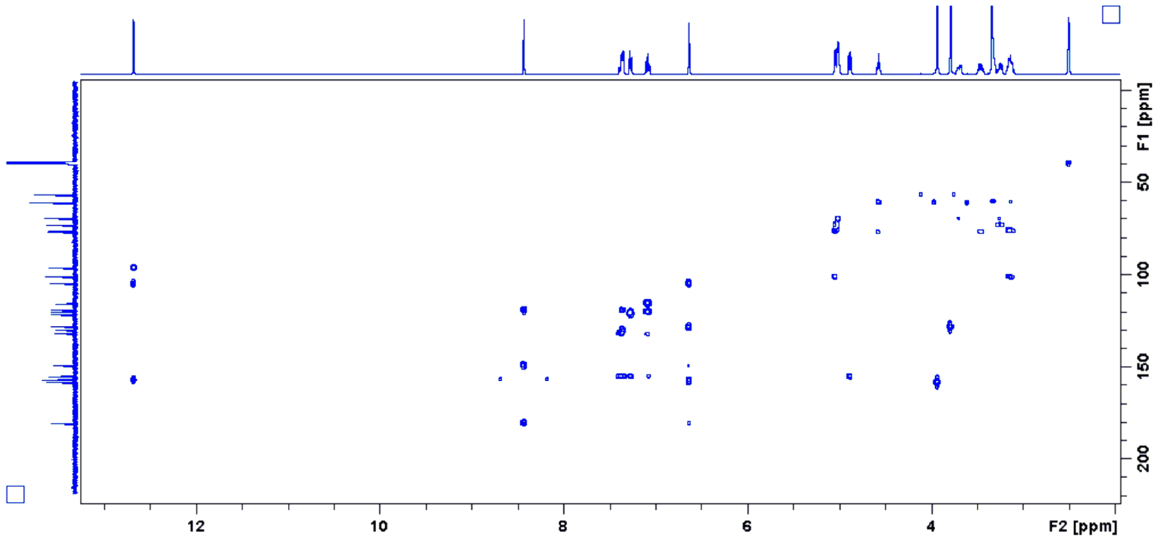

Figure S30 HMBC spectrum of compound **9** (DMSO-d_6)

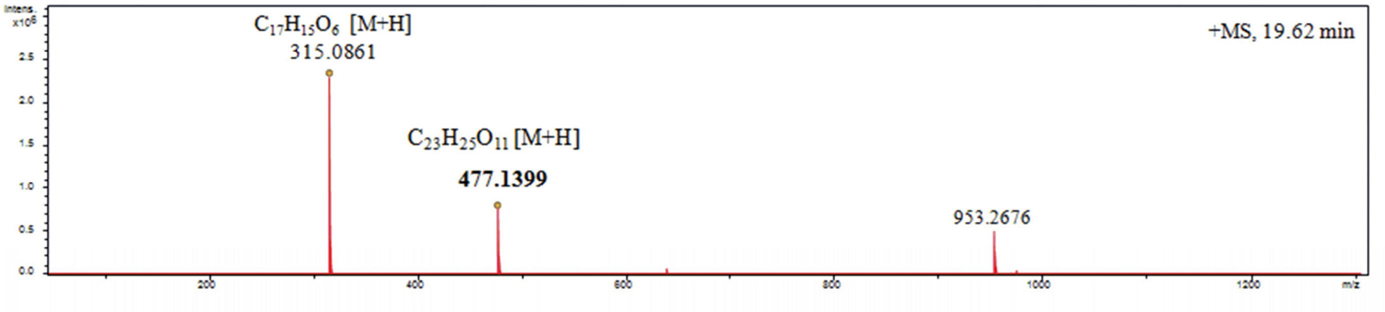

Figure S31 HR-ESI-MS spectrum of compound **9**

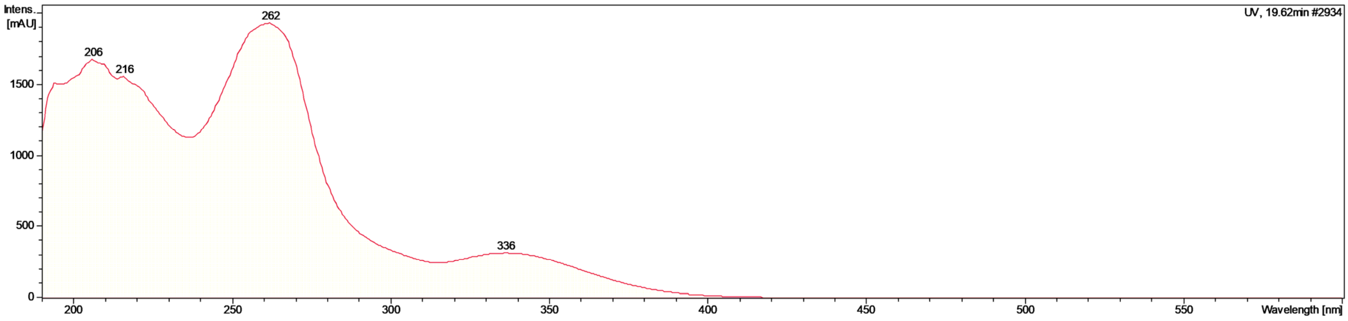

Figure S32 UV spectrum of compound **9**

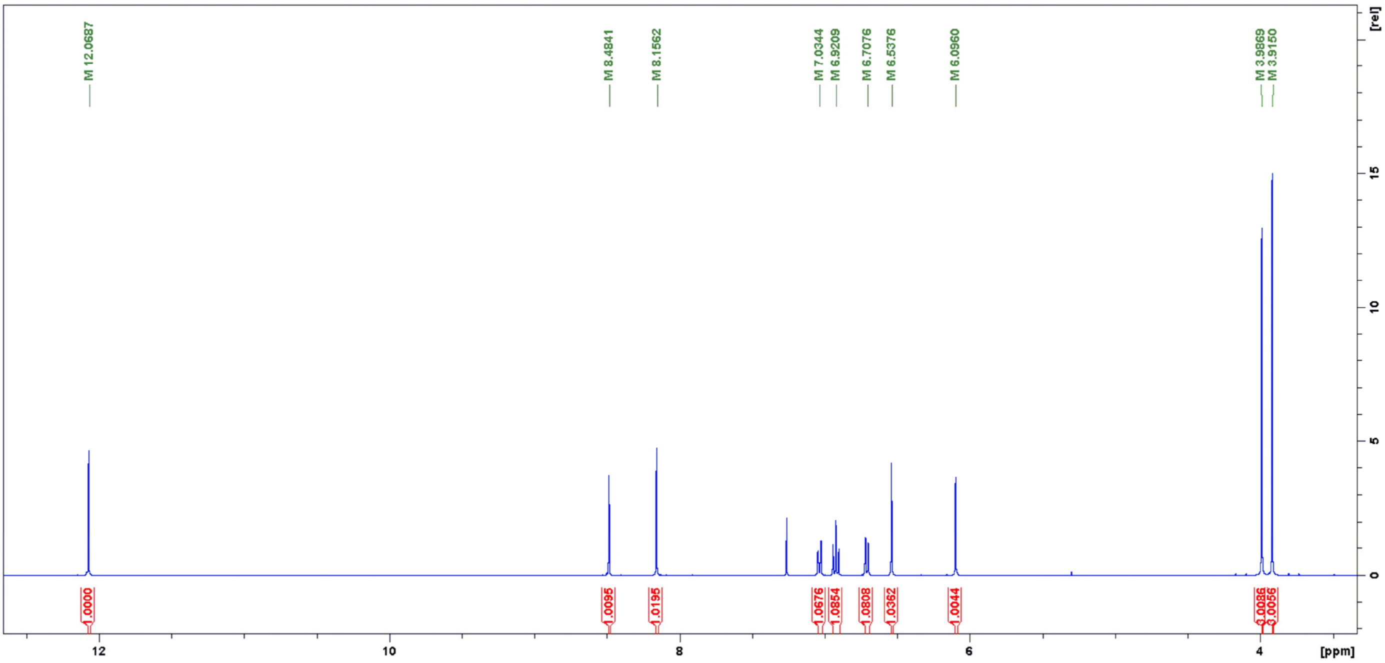

Figure S33 ^{1}H NMR spectrum of compound **10** (500 MHz, $CDCl_3$)

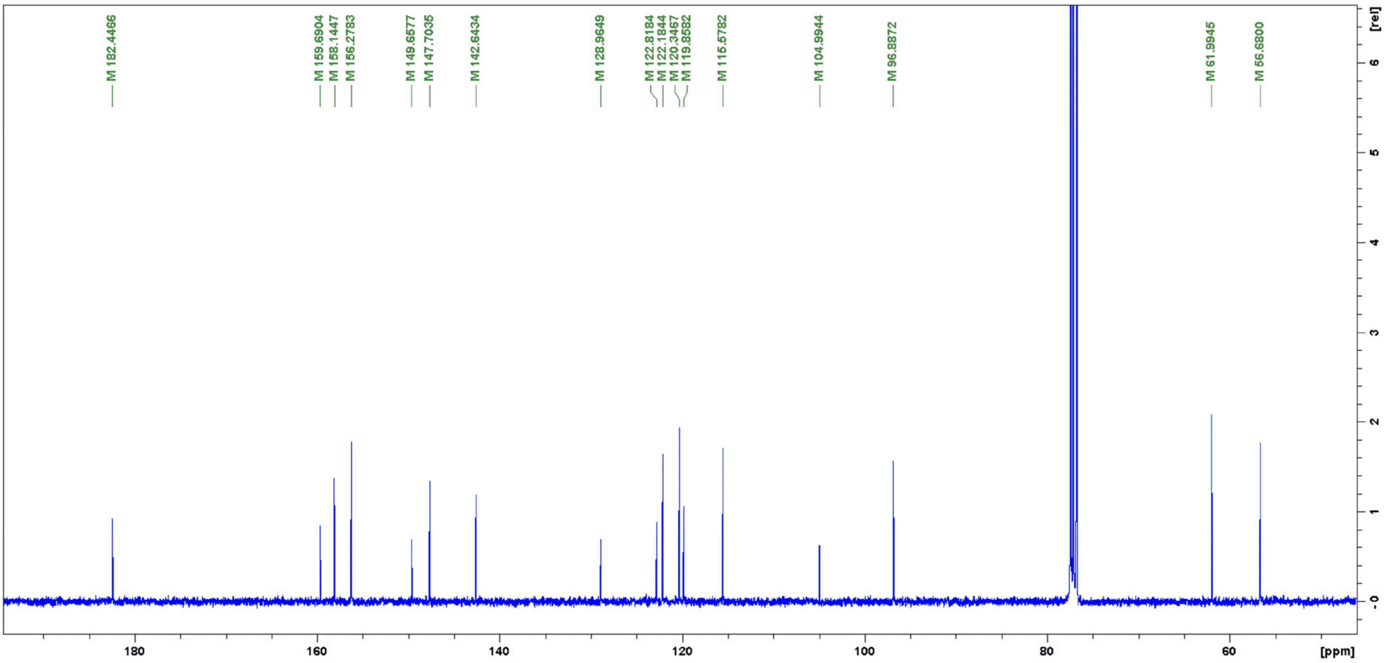

Figure S34 ^{13}C NMR spectrum of compound **10** (125 MHz, $CDCl_3$)

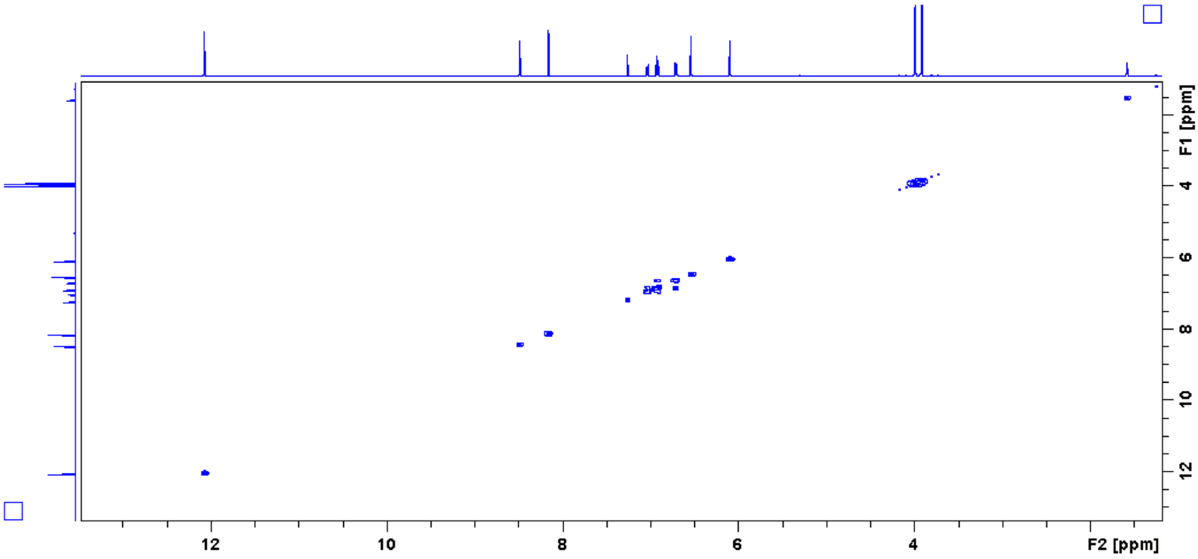

Figure S35 ^{1}H-^{1}H COSY spectrum of compound **10** ($CDCl_3$)

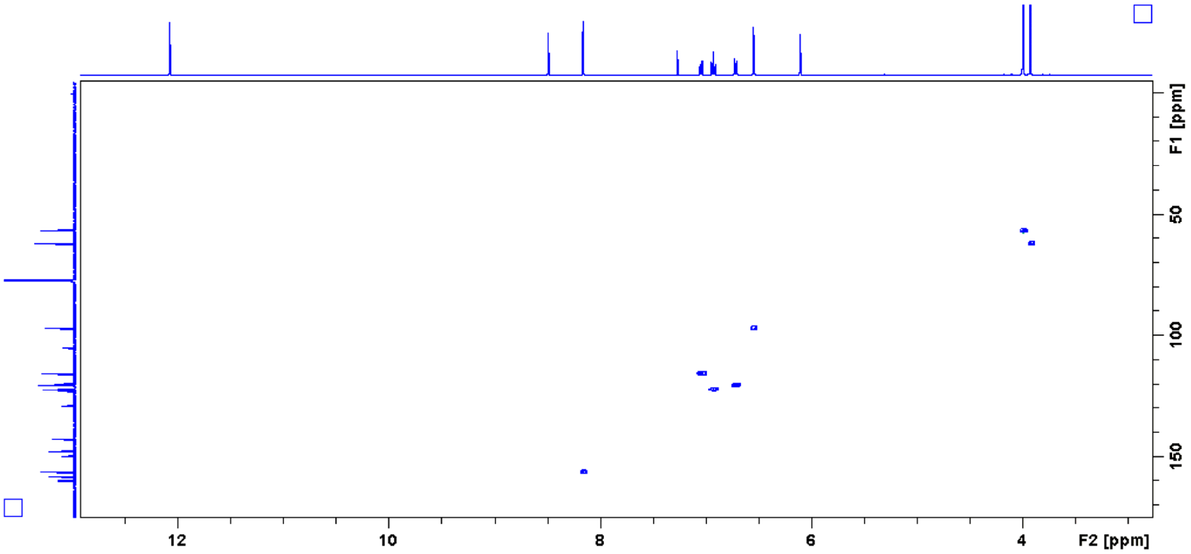

Figure S36 HSQC spectrum of compound **10** ($CDCl_3$)

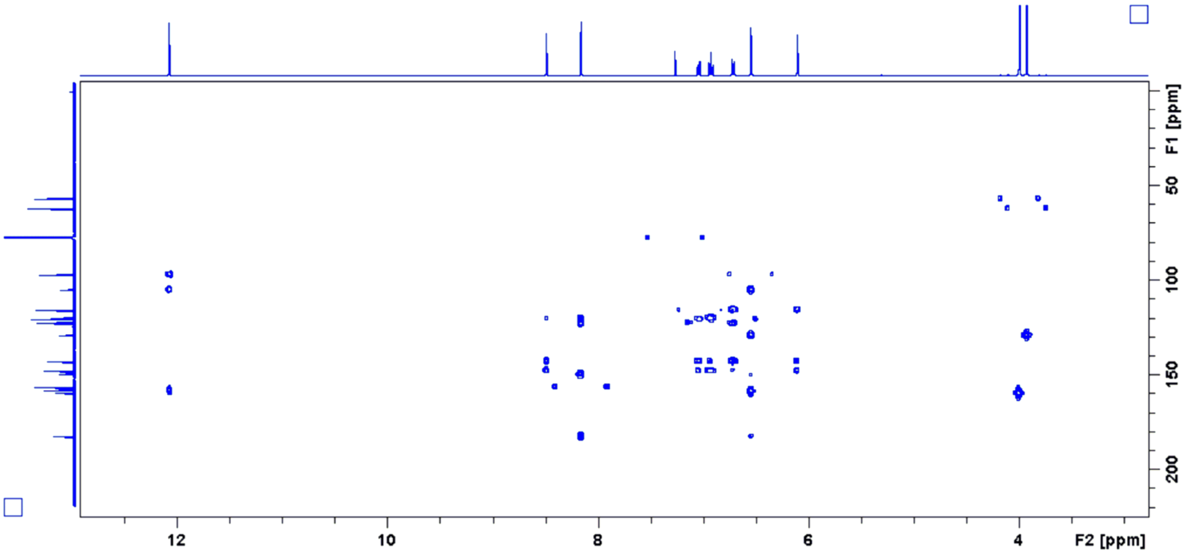

Figure S37 HMBC spectrum of compound **10** ($CDCl_3$)

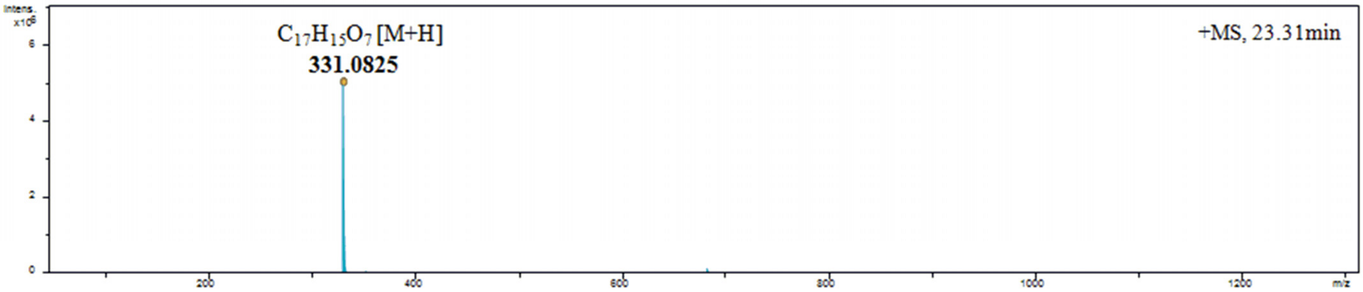

Figure S38 HR-ESI-MS spectrum of compound **10**

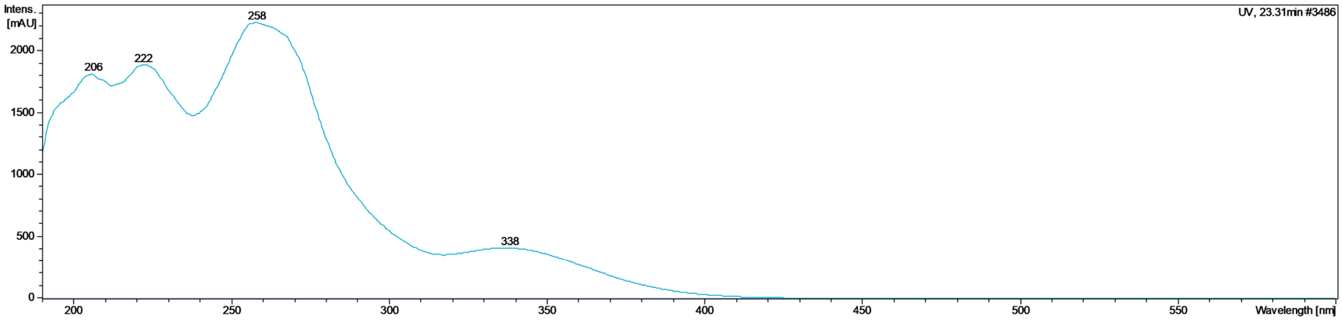

Figure S39 UV spectrum of compound **10**
